# Prophage ΦESI promotes competitive dominance in the emergent *Salmonella* serovar Infantis lineage

**DOI:** 10.1128/spectrum.01159-26

**Published:** 2026-09-25

**Authors:** Cristobal Martinez-Padilla, Daniel Tichy-Navarro, Rocio Barron-Montenegro, Ignacia Herrera-Vergini, Juan A. Ugalde, Diana Álvarez-Espejo, Andrea I. Moreno-Switt, Alejandro Piña-Iturbe

**Affiliations:** 1 Escuela de Medicina Veterinaria; Facultad de Agronomía y Sistemas Naturales, Facultad de Ciencias Biológicas y Facultad de Medicina, Pontificia Universidad Católica de Chile 28033, Santiago, Chile; 2 Center for Bioinformatics and Integrative Biology, Facultad de Ciencias de la Vida, Universidad Andrés Bello 28087, Santiago, Chile; 3 Instituto de Ingeniería Biológica y Médica, Facultad de Ingeniería, Facultad de Ciencias Biológicas y Facultad de Medicina, Pontificia Universidad Católica de Chile 28033, Santiago, Chile; 4 Escuela de Enfermería, Facultad de Medicina, Pontificia Universidad Católica de Chile 28033, Santiago, Chile; Universidad Tecnica de Ambato, Ambato, Ecuador; University of Hertfordshire, Hatfield, Hertfordshire, United Kingdom

**Keywords:** prophage, *Salmonella* Infantis, pESI megaplasmid, pathogen emergence

## Abstract

The global emergence of multidrug-resistant *Salmonella enterica* serovar Infantis has been largely attributed to the pESI megaplasmid; however, additional factors contributing to the ecological success of this lineage remain poorly understood. Here, we characterize a phage of emergent *Salmonella* Infantis (ΦESI), a temperate bacteriophage discovered during coculture of an emergent pESI-positive strain (PM57) and a non-emergent strain (DR006). Phage ΦESI shows a siphovirus morphotype with a long tail and an elongated head, with a genome of 46,490 bp, which is integrated as a prophage at the 3′-end of an Arg-tRNA gene in PM57, but is absent in DR006, which is susceptible to ΦESI-mediated lysis. Screening of ΦESI genes across 20,429 global *Salmonella* Infantis genomes revealed a near-exclusive association of ΦESI and ΦESI-like prophages with the emergent pESI-positive lineage. Further bioinformatic analyses of complete chromosomes revealed diverse ΦESI insertion profiles showing geographic clustering and the presence of large-scale chromosomal inversions flanked by ΦESI genes. *In vitro* competition assays demonstrated that PM57 outcompeted DR006 in coculture, coinciding with high viral loads that selectively lysed DR006. Consistently, susceptibility assays showed that strains lacking ΦESI/ΦESI-like prophages were susceptible to ΦESI infection. Together, these findings identify ΦESI as a lineage-associated prophage that mediates competitive exclusion of susceptible strains. Our results support a role for prophage-driven competition in the ecological success of the multidrug-resistant *Salmonella* Infantis lineage.

**IMPORTANCE:**

Emergent multidrug-resistant *Salmonella enterica* serovar Infantis strains carrying pESI megaplasmids have spread worldwide, posing a global public health threat and a significant economic burden. Nevertheless, the factors contributing to the success of this foodborne pathogen, beyond pESI, remain poorly understood. Here, we describe ΦESI, a novel temperate bacteriophage carried by the emergent lineage as a chromosomally integrated prophage. We show that ΦESI provides a competitive advantage to emergent strains by selectively killing non-emergent competitors while protecting lysogens from reinfection. Our findings uncover the contribution of ΦESI/ΦESI-like bacteriophages to the success of emergent *Salmonella* Infantis, highlighting how lineage-associated prophages can shape the ecological success of pathogenic bacteria.

## INTRODUCTION

Non-typhoidal *Salmonella* (NTS) is a major cause of foodborne disease worldwide, affecting millions of people and causing approximately 215,000 deaths annually (1). Usually producing self-limiting gastroenteritis, NTS can also cause a severe invasive infection requiring antibiotic treatment, including the use of third-generation cephalosporins, fluoroquinolones, and azithromycin (2). While more than 2,600 *Salmonella* serovars have been identified, most human cases are caused by a limited number of highly prevalent serovars (3–5).

Over the past two decades, multidrug-resistant (MDR) and extended-spectrum β-lactamase (ESBL)-producing *Salmonella* enterica serovar Infantis have emerged as a global zoonotic pathogen primarily associated with consumption of chicken meat, likely shaped by international poultry trading (6, 7). The success of emergent *Salmonella* Infantis is mainly linked to the acquisition of a ≈300 Kbp pESI/pESI-like megaplasmid carrying multiple antimicrobial resistance genes [e.g., *aph(3’)-Ia*, *aph(4)-Ia*, *aac(3)-Iva*, *floR*, *fosA3*, *sul1*, *tet*(A), *bla*_CTX-M-1_, and *bla*_CTX-M-65_] along with virulence (e.g., yersiniabactin synthesis locus, K88-like fimbria, and Infantis plasmid-encoded fimbria), disinfectant-resistance (*qacEΔ1*), and heavy-metal tolerance (e.g., *mer* operon) determinants (8–13). Moreover, chromosomal mutations in the quinolone resistance-determining region of the *gyrA* gene are common in the emergent *Salmonella* Infantis lineage (7). Together, these acquired features contribute to enhanced chicken gut colonization, increased shedding, persistence in chicken meat processing facilities, and resistance to multiple antibiotics, including those used in chicken farms and as first-line treatment for severe infections in humans (8, 9, 14–17). In addition, the widespread dissemination of emergent *Salmonella* Infantis across all continents (except Antarctica) and among multiple environments and niches, including surface/irrigation waters, wild animals, livestock, companion animals, poultry, and food, makes this MDR pathogen a major threat to global public health (7, 18–20). While strong evidence supports pESI-like megaplasmids as major contributors to the successful dissemination of emergent *Salmonella* Infantis, the impact or potential role of other genetic factors remains unexplored.

Bacteriophages (phages), highly diverse viruses that infect bacteria, are major contributors to bacterial diversification, shaping their genetic content and ecological interactions (21). They replicate primarily through two cycles: the lytic cycle, in which active phage replication leads to the production, assembly, and release of new viral particles, lysing the host cell (22), and the lysogenic cycle, in which the phage genome integrates into the bacterial chromosome as a prophage and is passively inherited during cell division (23). Phages that undergo only the lytic cycle are termed virulent or strictly lytic phages, while temperate phages can follow either the lytic or lysogenic pathway (23). During lysogenic infection, the temperate phage genome integrates into the bacterial chromosome either randomly or at specific “attachment sites” (*att* sites), through a reaction catalyzed by integrases encoded by the phage genome (24, 25). The lysogenic state is maintained by transcriptional repressors encoded by lysogenic master regulator genes within the prophage genome; these repressors inhibit the expression of lytic genes such as holins and lysins (24). Prophages can be reactivated through induction by SOS-dependent or SOS-independent mechanisms, including exposure to DNA-damaging agents (e.g., UV radiation), reactive oxygen species, quorum-sensing signals, phage-encoded regulatory systems, phage–phage interactions, or spontaneous induction (24, 26–30).

Prophages are common in clinically and environmentally important bacteria, and they dominate the virome in the gut-associated microbiomes (31–33). They influence community structure through lysogeny, lysis, and horizontal gene transfer, enhancing bacterial genetic flexibility and adaptation (33, 34). Prophages can also provide competitive advantages to their bacterial hosts against susceptible subpopulations. For example, the Lambda prophage can benefit its *Escherichia coli* hosts, acting as a “replicative toxin” against susceptible bacteria, improving the survival of its carriers in both solid and liquid environments (35). Similarly, *Enterococcus faecalis* V583 uses phage particles to maintain dominance in its intestinal niche (36). Moreover, in *Salmonella* Typhimurium, spontaneous induction of the Gifsy prophage provides a strong growth advantage in coculture, underscoring how prophage activation can decisively influence bacterial competition (37).

Only a few comparative studies have assessed the prophage content of *Salmonella* Infantis among emergent and non-emergent isolates. Although these works were limited by relatively small genome data sets, they showed evidence of specific prophages that correlate with phylogenetic clades of *Salmonella* Infantis and were unique to emergent genomes (38, 39), suggesting a potential role of these prophages in the diversification of this foodborne pathogen. In this study, we describe the isolation and characterization of a phage of emergent *Salmonella* Infantis (ΦESI), a temperate bacteriophage associated with the emergent *Salmonella* Infantis lineage, providing compelling evidence that it confers a competitive advantage over non-emergent strains lacking this prophage.

## RESULTS

### ΦESI is a lysogenic bacteriophage integrated into the chromosome of pESI-positive *Salmonella* Infantis PM57

**Fig 1:**
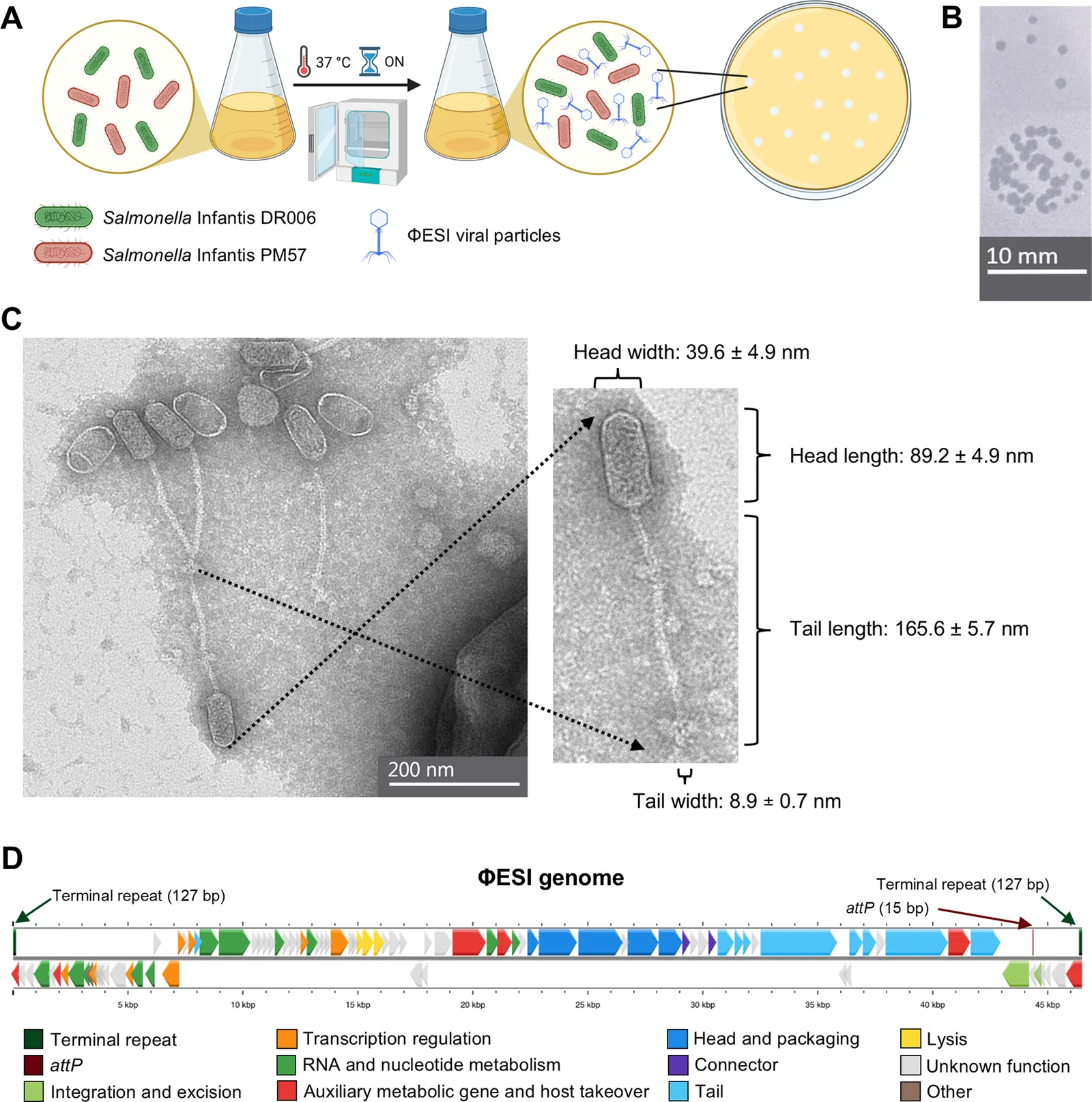
Isolation, phenotypic, and genotypic characterization of ΦESI**.** (**A**) Schematic representation of the experimental procedure used to isolate phage ΦESI, where *Salmonella* Infantis PM57 (red), from the emergent lineage, and the non-emergent isolate DR006 (green) were cocultured (ON: overnight) on trypticase soy broth media, and the filtered supernatant used for phage isolation. (**B**) Lysis plaque morphology of ΦESI on a double-layer agar of *Salmonella* Infantis DR006. A bar scale of 10 mm is shown. (**C**) Transmission electron micrograph of the ΦESI bacteriophage. The tail and head length and width (mean ± standard deviation) were estimated from measurements of seven viral particles. (**D**) Linear representation of the ΦESI phage genome. Genes were colored according to their predicted function as determined by the Pharokka annotation. The 127 bp terminal repeats and the 15 bp *attP* site are indicated with arrows.

The bacteriophage ΦESI was isolated from a coculture of *Salmonella* Infantis strain PM57 (pESI-positive; emergent lineage) and strain DR006 (pESI-negative; non-emergent lineage; Fig. 1A). ΦESI produced clear lytic plaques approximately 1 mm in diameter on double-layer agar plates using DR006 as the host strain (Fig. 1B; Fig. S1 see Data Availability section for Supplementary Files at https://doi.org/10.6084/m9.figshare.31821361). Transmission electron microscopy (TEM) revealed that ΦESI exhibits a siphovirus morphotype, characterized by a long, flexible tail (165.6 ± 5.7 nm) and an elongated head structure (length: 89.2 ± 4.9 nm; width: 39.6 ± 4.9 nm; Fig. 1C; see Fig. S2 and Table S1 at https://doi.org/10.6084/m9.figshare.31821361). The genomic analysis showed that ΦESI has a 46,490 bp genome with a 49.3% G + C content and 88 coding sequences (CDSs) according to the Pharokka annotation. The genome was flanked by 127 bp long direct terminal repeats (TTTGTTTTTACCGGCTGCTTTCCATGCAGTGAATGCGCCGTTGTCCAGCGCGAATGACTGGCAGTATTCAGCCGCGAGGTTAATTTGTCCGGAATGCGCGAAACTGATAAACGCATGGCGTCCTTTC) and was predicted to encode several lysogeny-associated proteins, including an integrase, suggesting a lysogenic lifestyle (Fig. 1D; see Table S2 at https://doi.org/10.6084/m9.figshare.31821361). A BLASTn search of the NCBI Virus nucleotide database using the genome of ΦESI yielded hits with ≤51% coverage. The two phage genomes showing the highest nucleotide identity had coverage values of 24% (phage Inf 5–2, 99% identity; GenBank accession OR850760.1) and 51% (phage FSL SP-016, 97% identity; GenBank accession KC139516.1). These results, together with the taxonomic analyses performed using VICTOR, VIRIDIC, and the top eight phage genomes from the NCBI Virus search, indicated that ΦESI may represent a novel bacteriophage genus within the class *Caudoviricetes* (see Fig. S3 at https://doi.org/10.6084/m9.figshare.31821361).

**Fig 2:**
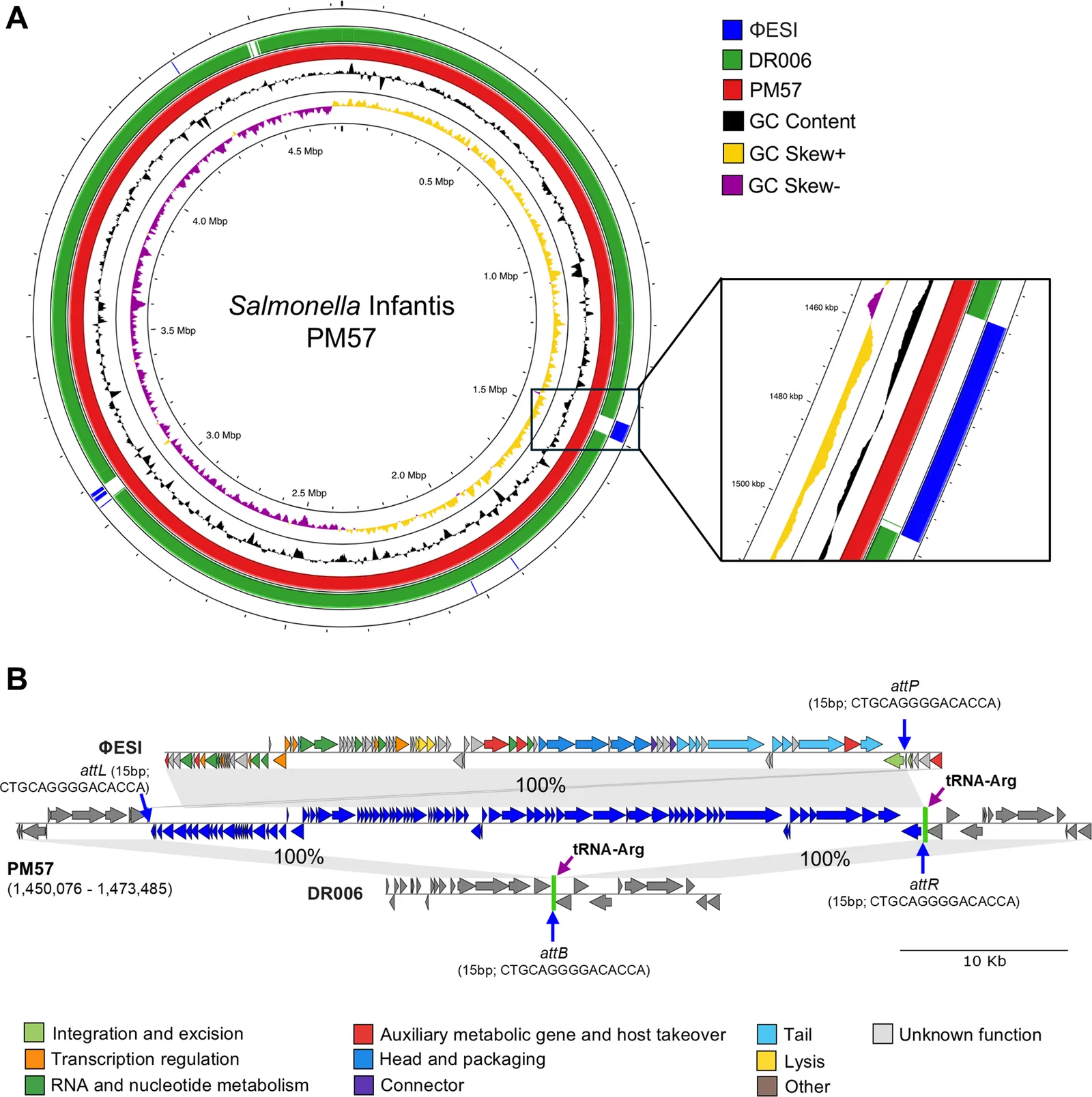
ΦESI is integrated as a prophage in the chromosome of *Salmonella* Infantis PM57. (**A**) Circular representation of the complete *Salmonella* Infantis PM57 chromosome (red) and the BLAST alignments of DR006 (green), and the genome of bacteriophage ΦESI (blue). A zoom-in view of the region where ΦESI is integrated into the PM57 chromosome is also presented. (**B**) BLAST alignment between the bacteriophage ΦESI genome, the prophage ΦESI region integrated into the chromosome of PM57 strain, and the corresponding region in strain DR006. Genes were colored according to their predicted function as determined by the Pharokka annotation using the same color palette as in Fig. 1D. The insertion site at the Arg-tRNA and the 15 bp attachment sites (*attP* in the virion DNA, *attL*/*R* flanking the prophage in PM57, and *attB* in DR006) are indicated with purple and blue arrows, respectively.

To assess the source of ΦESI, BLASTn alignments were carried out against the genomes of the two cocultured strains, PM57 and DR006. The analysis revealed the presence of ΦESI as a prophage integrated within the chromosome of strain PM57 at the 3′-end of an Arg-tRNA-encoding gene, spanning positions 1,451,172–1,497,549 (Fig. 2), while it was absent from the genome of strain DR006 (Fig. 2A). The integrated prophage was flanked by 15 bp long direct-repeated sequences (left and right attachment sites; *attL* and *attR*) corresponding to the last nucleotides of the tRNA gene (CTGCAGGGGACACCA; Fig. 2B). An exact match of these sites was present in the virion genome at position 44,353, located 164 bp upstream of the integrase gene, representing the *attP* site. In addition, the 127 bp long terminal repeats flanking the virion genome were present in the prophage as a single internal copy at position 1,453,183. Although strain PM57 and DR006 encode 7 Arg-tRNAs, the one harboring the ΦESI integration site is unique in sequence, including the 15 bp long sequence (*attB* site) which in strain DR006 (the *attB* site) is not occupied (Fig. 2B). Consistent with these findings, ΦESI formed lytic plaques on DR006, whereas the strain PM57 was resistant to plaque formation (see Fig. S4 at https://doi.org/10.6084/m9.figshare.31821361). Our analyses confirmed that the isolated bacteriophage came from the genome of strain PM57.

### ΦESI and ΦESI-like prophages are associated with emergent *Salmonella* Infantis and major chromosome rearrangements

**Fig 3:**
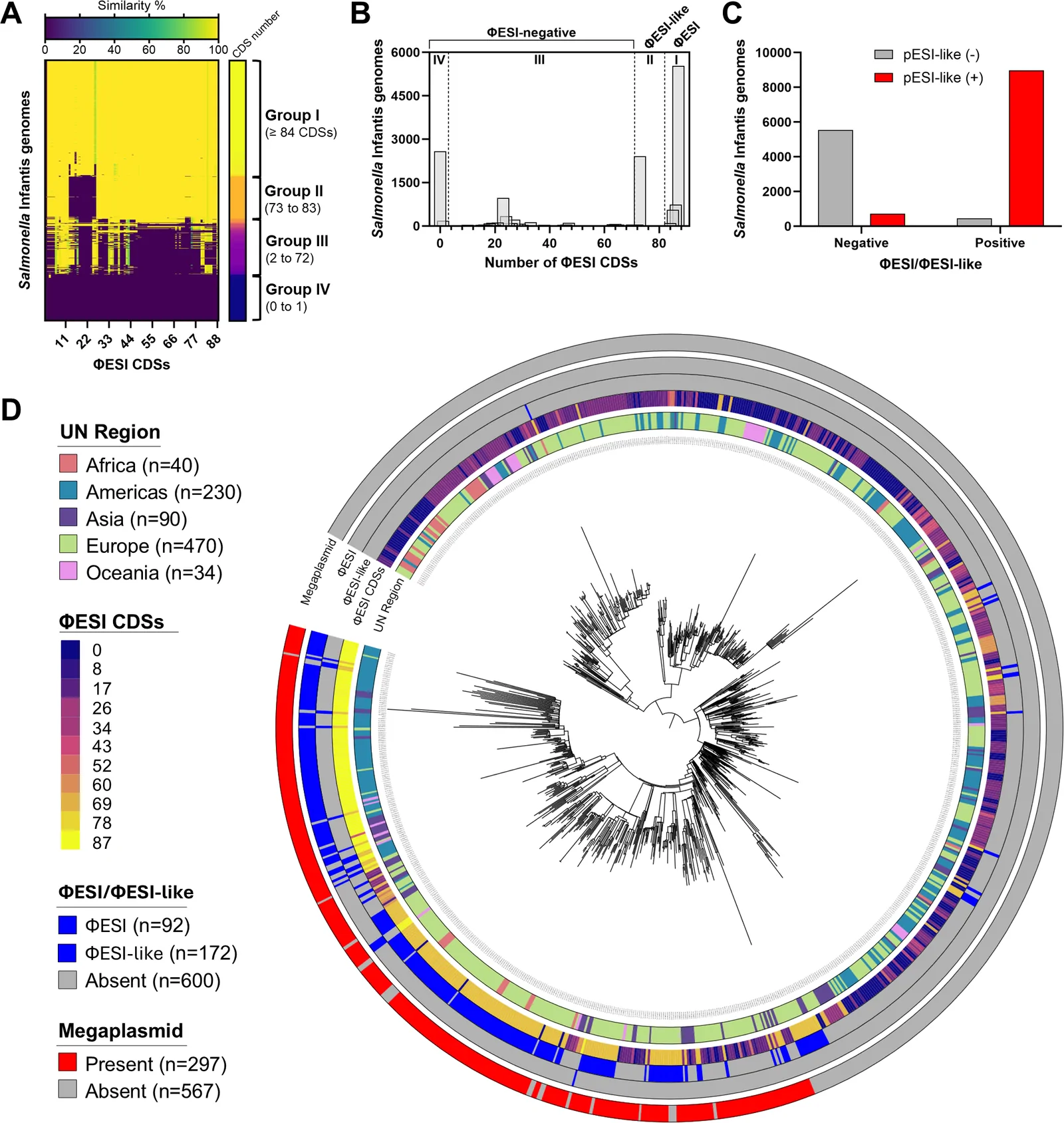
ΦESI and ΦESI-like prophages are associated with pESI-positive *Salmonella* Infantis. (**A**) Heatmap depicting the presence and similarity of the ΦESI CDSs across 15,707 non-clonal *Salmonella* Infantis genomes representing the global diversity. The CDSs are ordered as in the ΦESI genome (from CDS_0001 to CDS_0088). The total number of ΦESI CDSs per bacterial genome is also indicated, together with the four groups (I–IV) in which the genomes were classified. (**B**) Histogram showing the distribution of the global 15,707-genome data set based on the number of ΦESI CDSs. Genome groups I–IV are also indicated. Group I and II genomes were considered to harbor ΦESI and ΦESI-like prophages. (**C**) Frequency of pESI-positive or -negative *Salmonella* Infantis genomes in the global data set categorized by the presence of ΦESI or ΦESI-like prophages. (**D**) Recombination-free core-SNP maximum likelihood phylogeny of 864 *Salmonella* Infantis genomes representing the global geographic, temporal, and genomic diversity of *Salmonella* Infantis from the HC200_36 cluster. The tree is rooted at the best-fitting temporal root. The rings show, from the inside to the outside, the geographic origin of the genomes at the continental (UN region) level, the number of ΦESI CDSs found in those genomes, the presence of ΦESI-like and ΦESI prophages, and the presence of pESI-like megaplasmids.

To assess the global distribution of the ΦESI prophage, we conducted bioinformatic and phylogenetic analyses based on a non-redundant 20,429-genome data set representing the global genomic, isolation source, temporal, and geographic diversity of *Salmonella* Infantis as of 9 September 2025. We searched for the 88 ΦESI CDSs (80% coverage and identity cutoffs) within a subset containing non-clonal genomes only (*n* = 15,707). We found variable conservation of the CDSs, revealing four major groups according to their presence/absence patterns and percent similarity (id.×cov./100; Fig. 3A and B; see Tables S3 and S4 at https://doi.org/10.6084/m9.figshare.31821361). Group I represented 44.1% (6,928/15,707) of non-clonal genomes and carried ≥84 ΦESI CDSs (mean similarity: 99.20%–99.99%); group II comprised 16.0% (2,514/15,707) of genomes and carried 73–83 CDSs (mean similarity: 98.70%–99.99%); group III comprised 22.3% (3,505/15,707) and carried 2 to 72 CDSs (mean similarity: 89.69%–100.00%); and group IV comprised 17.6% (2,760/15,707), carrying 0–2 ΦESI CDSs (mean similarity: 85.19%–97.76%). Based on this distribution, we classified the genomes into three categories: group I as ΦESI-positive; group II as ΦESI-like-positive; and groups III–IV as ΦESI-negative genomes. According to this grouping, a statistically significant association (*χ*², *P* < 0.0001) was found between the presence of ΦESI and ΦESI-like prophages and the emergent lineage of *Salmonella* Infantis, represented by the carriage of pESI-like megaplasmids. In particular, 92.5% (*n* = 8,978/9,706) of emergent genomes harbored the prophages compared with only 7.7% (*n* = 464/6,001) of non-emergent genomes (Fig. 3C and D). The odds ratio computed from the contingency table was 147.2 (95% CI: 130.4–166.1), indicating that plasmid-carrying genomes had ~147-fold higher odds of prophage carriage than genomes lacking the plasmid. Together, these findings suggest that ΦESI-like prophages are tightly linked to the globally emergent pESI-positive *Salmonella* Infantis lineage.

**Fig 4:**
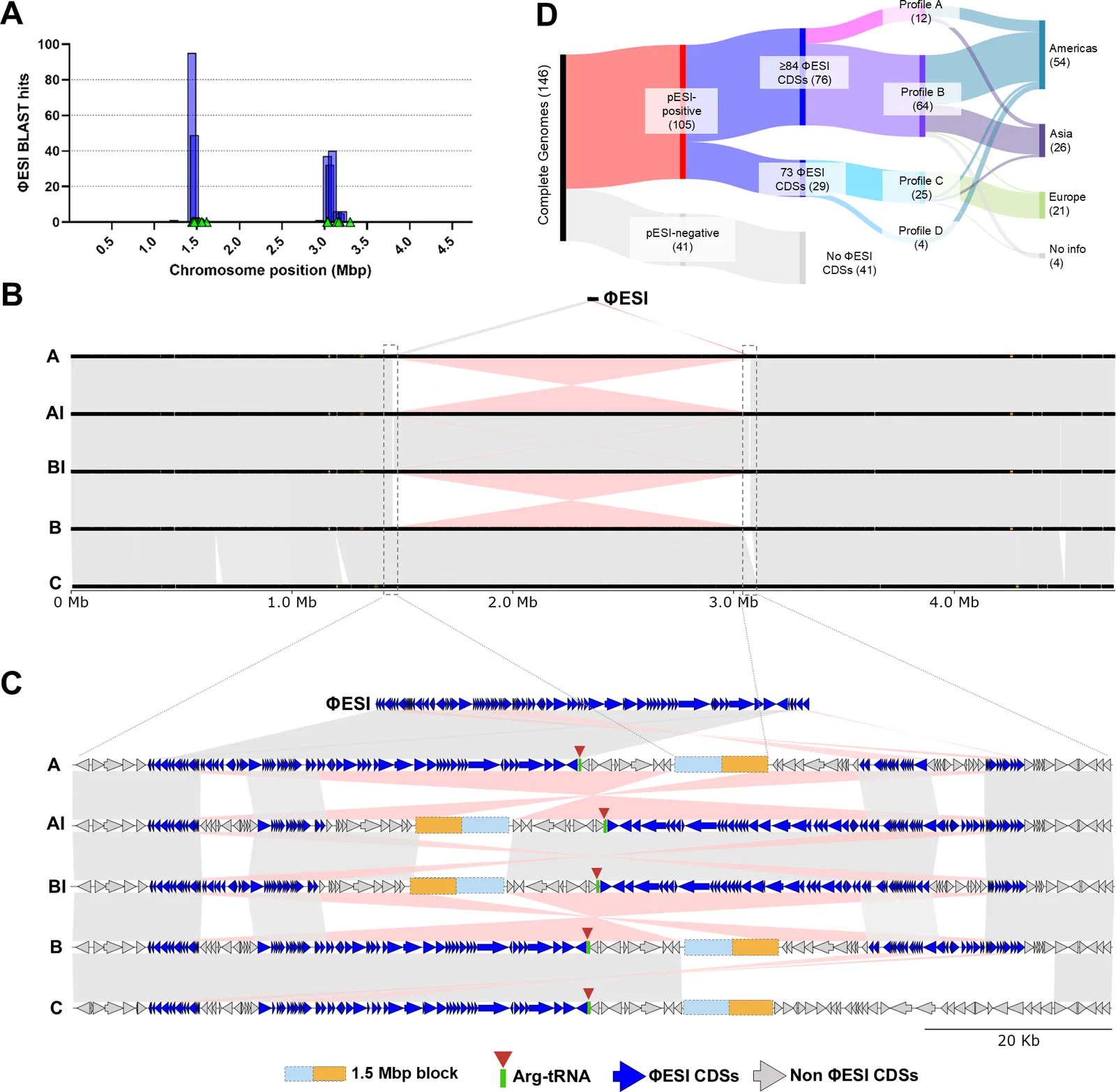
ΦESI and ΦESI-like prophages are found at conserved chromosome locations and are related to large chromosomal inversions. (**A**) Histogram showing the distribution of ΦESI-derived ABRicate hits across chromosomal coordinates among 105 *Salmonella* Infantis closed genomes. The green triangles indicate the location of the 15 bp long *attB* site. (**B**) BLAST alignment of the chromosome of representative isolates with different profiles of ΦESI insertion showing a large-scale chromosomal inversion flanked by the ΦESI/ΦESI-like insertion sites. BLAST hits (>99% identity) are represented as gray and pink lines connecting regions with direct and inverse sequence identity, respectively. (**C**) Zoom-in view of the BLAST alignment of representative isolates in panel **B**. ΦESI CDS are colored in blue, while non-ΦESI CDS are colored in gray. BLAST hits (>99% identity) are represented as gray and pink lines connecting regions with direct and inverse sequence identity, respectively. The associated Arg-tRNA is represented as a green line with a red arrow, and the 1.5 Mbp region between insertion positions is represented as blue and orange boxes. (**D**) Flow diagram describing the distribution of characteristics of the 146 *Salmonella* Infantis complete genomes used in these analyses.

Then, as the BLASTn search of ΦESI in PM57 revealed the presence of incomplete ΦESI sequences around position 3.0 Mbp (Fig. 2A), we assessed the chromosomal location of ΦESI sequences across 146 closed *Salmonella* Infantis chromosomes available in the NCBI Pathogen Detection database (see Table S5 at https://doi.org/10.6084/m9.figshare.31821361). To enable position-based comparisons, chromosome sequences were rotated or reoriented, if needed, to start at the *dnaN* gene and match the orientation of the N55391 reference. Our screening detected ΦESI-related sequences (≥5% coverage and ≥80% identity) in 105/146 (71.9%) genomes, all of which were pESI positive, exhibiting different completeness patterns (see Table S6 at https://doi.org/10.6084/m9.figshare.31821361). Notably, the sequences were found in two chromosomal loci located at positions ≈1.45 Mbp and ≈3.05 Mbp, delimiting a ≈1.5 Mbp intervening region (Fig. 4A). Overall, 25/105 ΦESI-positive genomes (23.8%) contained ΦESI-related sequences at or near the 1.45 Mbp locus, and 80/105 (76.2%) displayed hits at both loci. No *Salmonella* Infantis chromosome harbored ΦESI sequences at the 3.05 Mbp locus only. Although each of the 146 analyzed chromosomes harbored a single copy of the 15 bp *attB* site, the site’s location varied between the 1.45 Mbp and 3.05 Mbp loci (Fig. 4A). This was concurrent with a large-scale chromosomal inversion of the intervening region between the 1.45 and 3.05 loci, present in 39.0% (41/105) of the ΦESI-positive genomes (Fig. 4B) when compared with the PM57 strain.

Based on these insertion patterns and their genomic context, the genomes positive for ΦESI sequences were classified into four insertion profiles: profile A (12/105, 11.4%), profile B (64/105, 61.0%), profile C (25/105, 23.8%), and others, assigned to profile D (4/105, 3.8%; Table 1 and Fig. 4C). Overall, profile A genomes contained a complete ΦESI prophage on one side of the ≈1.5 Mbp intervening region and ΦESI remnants on the other side, as observed in PM57. Profile B genomes harbored a ΦESI-like prophage on one side of the intervening region and ΦESI remnants on the other. Together, however, these two loci retained the complete repertoire of ΦESI CDSs. Both profiles A and B included genomes carrying the large-scale chromosomal inversion, which resulted in reciprocal changes in the positions of the ΦESI/ΦESI-like prophage and the ΦESI remnants. In contrast, profile C genomes harbored a ΦESI-like prophage only at the ≈1.45 Mb locus, with no additional ΦESI-related sequences detected elsewhere in the chromosome. Singleton variants with ΦESI remnants flanking the intervening region were grouped into profile D.

**TABLE 1:** ΦESI insertion profiles among 101 closed genomes of *Salmonella* Infantis

Insertion profile	No. of genomes	Representative isolate	Inversion	ΦESI fragment length (bp)	ΦESI coverage (%)	Start position	End position	Strand
A	8/101	PM57	No	44,366	95.43%	1,453,762	1,498,128	Plus
				7,776	16.73%	3,068,789	3,076,565	Minus
				3,723	8.01%	3,082,395	3,086,118	Minus
	4/101	FSIS12214921	Yes	3,723	8.01%	1,451,771	1,455,494	Plus
				7,777	16.73%	1,461,324	1,469,101	Plus
				44,366	95.43%	3,035,507	3,079,873	Minus
B	37/101	N55391	Yes	16,360	35.19%	1,451,779	1,468,139	Plus
				35,783	76.97%	3,034,552	3,070,335	Minus
				3,723	8.01%	3,076,165	3,079,888	Minus
	27/101	Z1324PSL0001	No	3,723	8.01%	1,451,534	1,455,257	Plus
				35,783	76.97%	1,461,087	1,496,870	Plus
				16,360	35.19%	3,063,284	3,079,644	Minus
C	25/101	CFSAN024778	No	3,723	8.01%	1,509,790	1,513,513	Plus
				35,783	76.97%	1,519,343	1,555,126	Plus

We observed evidence of phylogeographic structuring in the presence and profiles of ΦESI/ΦESI-like prophages in the analyzed genomic data sets. Analysis of 20,429 genomes representative of the global geographic, temporal, niche, and genomic diversity of *Salmonella* Infantis (EnteroBase; 9 September 2025) revealed that 94.1% (7,018/7,460) of the ΦESI-positive genomes came from the Americas (Fig. 3D). In accordance, analysis of the distribution of complete chromosome profiles (Fig. 4D) revealed that profile B (ΦESI-positive) genomes were enriched among isolates from the Americas (Fisher’s exact test, *P* = 0.014), and profile C (ΦESI-like) genomes were associated with Europe (Fisher’s exact test, *P* < 0.0001).

As ΦESI/ΦESI-like prophages were almost exclusively carried by the emergent *Salmonella* Infantis lineage, and ΦESI from strain PM57 showed lytic activity against the non-emergent DR006 strain, we hypothesized that ΦESI/ΦESI-like phages may provide a competitive fitness to their hosts over ΦESI/ΦESI-like-negative strains.

### ΦESI provides a competitive advantage to emergent *Salmonella* Infantis *in vitro*

**Fig 5:**
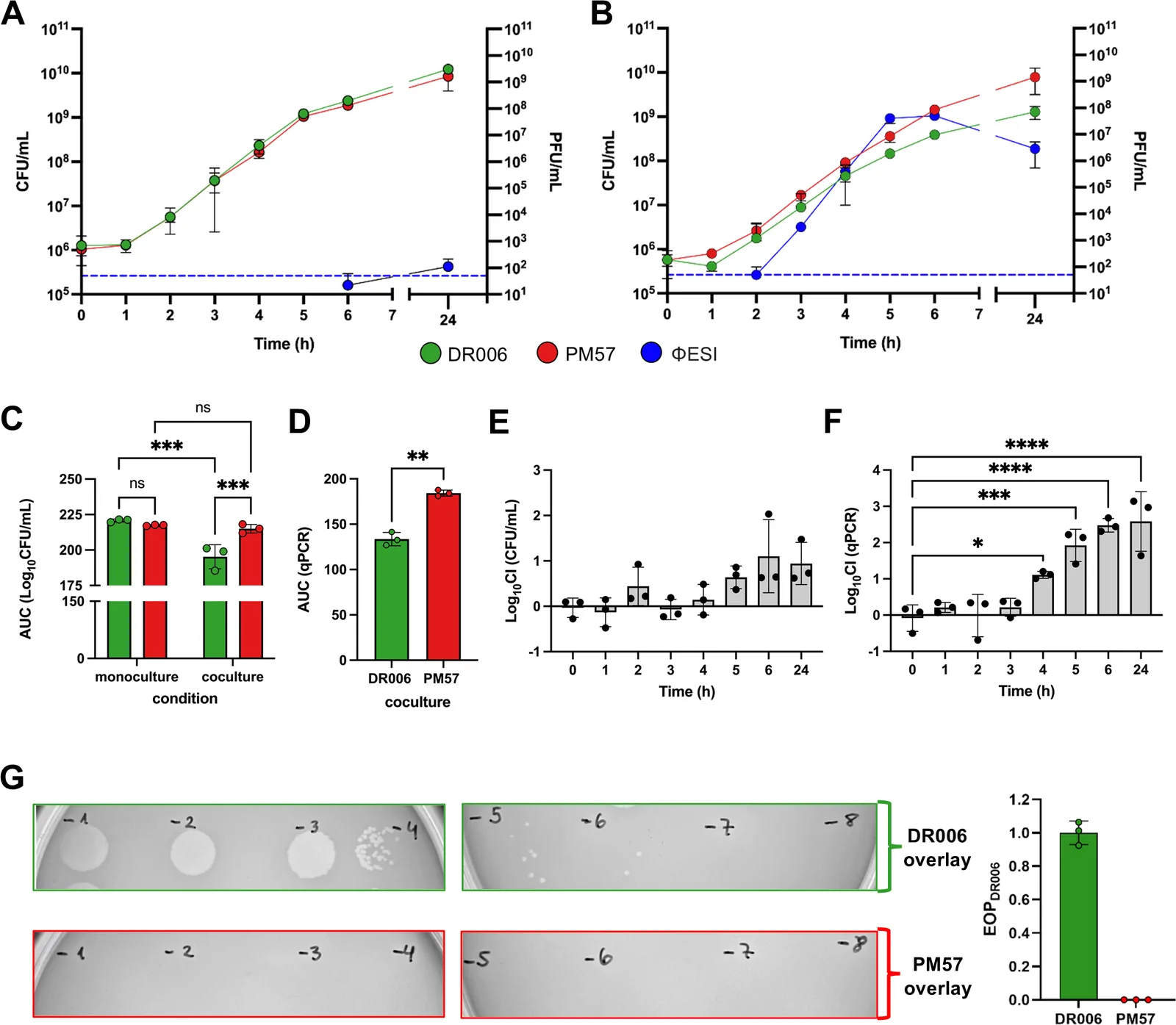
ΦESI-positive *Salmonella* Infantis PM57 has a competitive advantage over isolate DR006 lacking prophage ΦESI. Growth curves of *Salmonella* Infantis isolates PM57 and DR006 in TSB medium as (**A**) monocultures or (**B**) cocultures. The error bars represent the standard deviation of three independent biological replicates. For each time point, *Salmonella* CFU per milliliter and absolute qPCR-based quantification (see Fig. S5 at https://doi.org/10.6084/m9.figshare.31821361) were derived from the same sample aliquots. At each time point, the titer of phage particles present in the cultures was also determined. The broken blue line represents the limit of detection for phage quantification. (**C**) Area under the curve (AUC) of the log10-transformed growth curves shown in panels **A **and **B**. (**D**) AUC of the log10-transformed absolute counts of PM57 and DR006 from the experiments in A and B, as determined by qPCR. (**E**) The log10 competitive index (CI; PM57/DR006), estimated from the experiments in panel **B**, is shown based on the CFU titers and (**F**) qPCR absolute quantifications. (**G**) Sample phage titer quantification showing that lysis plaques formed only when isolate DR006 was used in the agar overlay. Quantification of the efficiency of plating relative to DR006 is also shown. See Materials and Methods for statistical details. Statistical significance thresholds were defined as follows: *P* < 0.05 (*), *P* < 0.01 (**), *P* < 0.001 (***), and *P* < 0.0001 (****).

Competition assays between PM57 (ΦESI-positive) and DR006 (ΦESI-negative) strains were carried out (see Table S7 at https://doi.org/10.6084/m9.figshare.31821361). When grown in monocultures, both strains displayed almost identical growth curves (Fig. 5A). However, in coculture, both strains grew following a similar pattern as in monocultures until 4 h only, followed by a reduction in the growth of strain DR006 which resulted in a difference of 0.63 log at 5 h, 0.82 log at 6 h, and 0.96 log at 24 h (Fig. 5B). Accordingly, while the area under the curve (AUC) of the two strains in monoculture did not differ statistically, in coculture, the AUC of DR006 was significantly lower than both the AUC of PM57 in coculture and the AUC of DR006 in monoculture (Fig. 5C; *P* < 0.001). The competitive index (CI) of PM57 relative to DR006 in coculture indicated that strain PM57 consistently exhibited higher fitness, as the CI increased above 0 over time despite both strains being inoculated at equal starting concentrations (Fig. 5E). This competitive advantage of strain PM57 became even more evident when both strains were quantified directly by strain-specific quantitative PCR (qPCR; see Fig. S5 at https://doi.org/10.6084/m9.figshare.31821361). Using this method, the AUC of strain PM57 in coculture remained significantly higher than that of strain DR006, confirming the previous observation (Fig. 5D). The CI calculated from absolute qPCR data further demonstrated a clear advantage of strain PM57, with statistically significant CI differences at 4, 5, 6, and 24 h compared with time 0 h (Fig. 5F). To quantify ΦESI during the competition assay, PFU/mL estimations on double-layer agar were performed at each time point for both monoculture and coculture samples (Fig. 5A and B; blue lines). In monoculture, low PFU levels were detected at 6 and 24 h for PM57, close to the assay’s detection limit (50 PFU/mL). No PFUs were detected in the monoculture of strain DR006. In contrast, in coculture, PFUs were detected as early as hour 2 and increased exponentially until hour 6, reaching a maximum of 5.04 × 10^7^ PFU/mL (Fig. 5B). The phage in the coculture formed lytic plaques exclusively on DR006 (Fig. 5G). These experiments indicate that the release of ΦESI infective particles from strain PM57 negatively impacts the growth of strain DR006, providing a competitive advantage under the coculture conditions.

**Fig 6:**
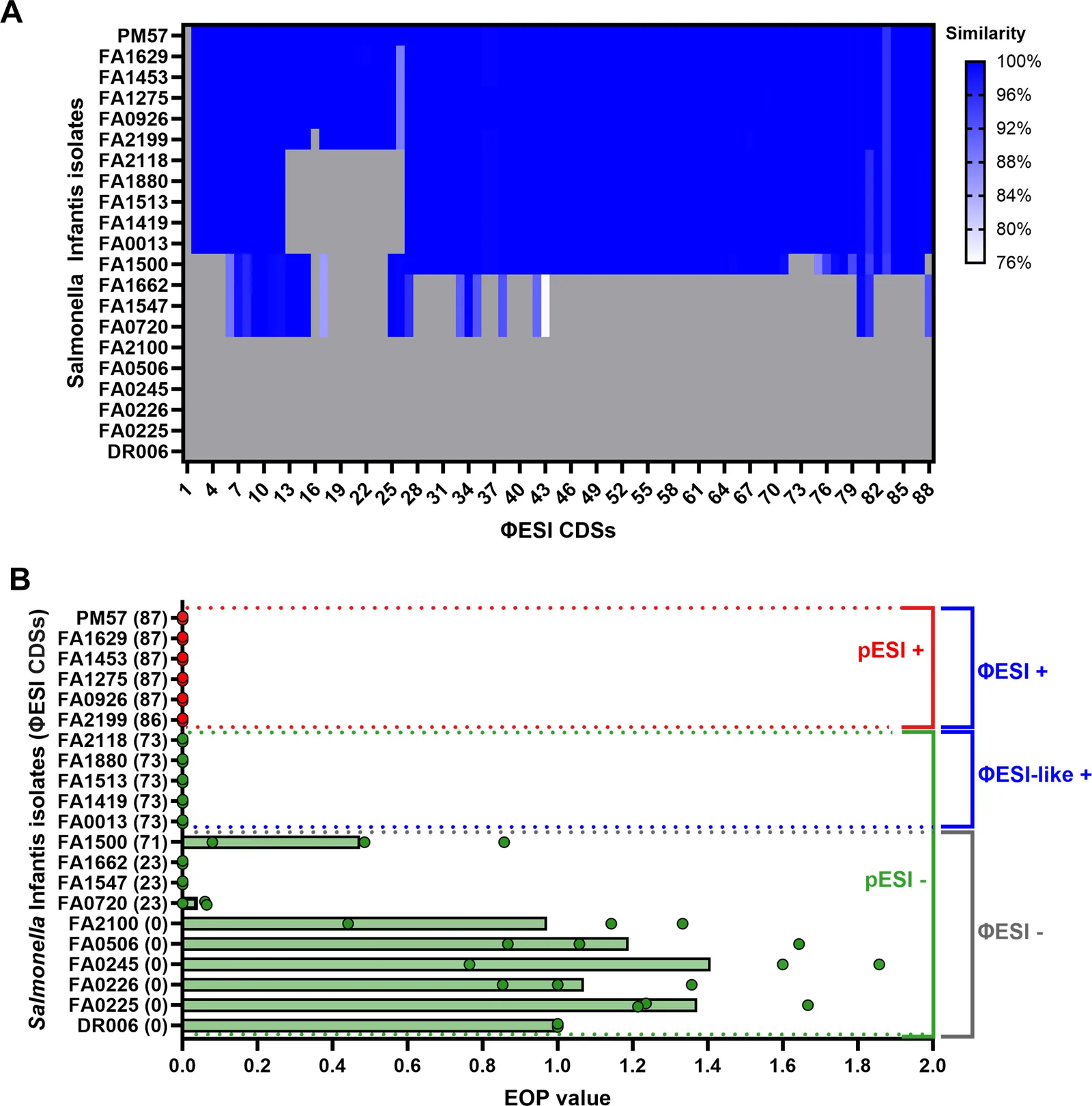
ΦESI and ΦESI-like prophages provide resistance against ΦESI-mediated lysis. (**A**) Heatmap showing the presence of ΦESI CDSs (from white to blue) among 21 *Salmonella* Infantis isolates used in the susceptibility to lysis assay. Gray color indicates the absence of a given CDS. The scale shows a similarity score based on the nucleotide identity and alignment coverage (see Materials and Methods for details). (**B**) The susceptibility of 21 different pESI-positive/negative and ΦESI/ΦESI-like positive/negative *Salmonella* Infantis isolates toward the ΦESI-mediated lysis. The horizontal bars represent the mean susceptibility expressed as the efficiency of plating using strain DR006 as the reference. The individual points indicate the EOP value for each of the three biological replicates performed. Strain PM57 was also included as a control. The Y axis indicates the different isolates tested and shows, in parentheses, the number of ΦESI CDSs present in each isolate. The red and green bars/points indicate the pESI-positive and pESI-negative isolates, respectively. The red and green brackets indicate the presence and absence of pESI-like megaplasmids. The blue and gray brackets indicate the presence and absence of ΦESI/ΦESI-like prophages.

We wanted to assess whether the observed interaction between PM57 and DR006 was not exclusive to these strains. Therefore, efficiency of plating (EOP) assays were carried out with 19 different *Salmonella* Infantis strains representing the diversity of ΦESI/ΦESI-like and pESI megaplasmid carriage found in our sequenced laboratory collection of 229 *Salmonella* Infantis isolates from Chile (see Table S8 at https://doi.org/10.6084/m9.figshare.31821361). The presence of ΦESI CDSs in these strains is shown in Fig. 6A. We could neither find pESI+/ΦESI-negative nor pESI+/ΦESI-like-positive combinations in our collection. After evaluating the susceptibility of the selected strains, we observed that all 10 ΦESI/ΦESI-like-positive isolates were resistant (EOP = 0) to the ΦESI-mediated lysis (Fig. 6B). Conversely, most (6/9) of the ΦESI-negative isolates were susceptible (EOP > 0.5). Among ΦESI-negative strains evaluated, those with zero ΦESI CDSs showed high values of EOP (about 1.0–1.4). On the other hand, in the strains carrying 71 and 23 ΦESI CDSs (FA1500, FA1662, FA1547, and FA0720; see Table S8 at https://doi.org/10.6084/m9.figshare.31821361) but still ΦESI-negative, the EOP was lower (FA1500, EOP = 0.5; 71 CDSs) or zero (FA1662, FA1547, and FA0720; 23 CDSs; Fig. 6B), suggesting a possible role for those genes in preventing ΦESI superinfection. Overall, the data indicate that *Salmonella* Infantis carrying ΦESI/ΦESI-like prophages have superinfection resistance, while non-carriers remain susceptible.

## DISCUSSION

The global dissemination of multidrug-resistant *Salmonella* Infantis represents a global economic burden and public health threat, further increased by the acquisition of ESBL-encoding genes (7). Carriage of the pESI megaplasmids and their variants, which confer enhanced colonization, persistence, and resistance features (8), has been recognized as the major contributor to the success of this foodborne pathogen, while the potential role of additional factors remained unknown.

Here, we discovered and characterized ΦESI, a prophage integrated into the chromosome of the emergent *Salmonella* Infantis PM57 strain, which can form active viral particles that infect and lyse the ΦESI-negative strain DR006, thereby providing a competitive advantage to its host. Previous research, on different bacterial species, has documented that prophage induction in a bacterial subpopulation can confer a competitive advantage to lysogenic bacteria by releasing infective phage particles that selectively lyse and kill susceptible non-lysogenic competitors, while the lysogenic population remains immune due to a superinfection exclusion mechanism (35, 37, 40, 41). Accordingly, our experiments showed the presence of phage-infective particles in the PM57 monoculture, while no decrease in the bacterial population was observed. This suggests a basal spontaneous induction of the prophage from a relatively small PM57 subpopulation. As we found no evidence that either nutrient depletion or a DR006-derived cue induced ΦESI (see Fig. S6 at https://doi.org/10.6084/m9.figshare.31821361), we therefore interpret the high phage titers observed in the coculture as resulting primarily from replication of ΦESI in the susceptible DR006 strain, which lacks all ΦESI CDSs. Of note, under the coculture experimental conditions, DR006 kept growing despite the presence of lytic phages in high titer. Whole-genome sequencing (WGS) of two independent lysis-resistant DR006 derivatives (SRA accession SRX34293353 and SRX34293354) discarded ΦESI integration and revealed one shared mutation only (c.468delA) in the *rfbB* (*wbaB*) gene resulting in a frameshift and premature stop (p.Lys156fs) of the encoded glycosyl transferase involved in the C1 serogroup O-antigen synthesis (42, 43) (see Fig. S7 at https://doi.org/10.6084/m9.figshare.31821361). Therefore, the Infantis lipopolysaccharide may constitute the ΦESI receptor as observed for multiple *Salmonella* phages (44).

In our experiments with different wild-type *Salmonella* Infantis, the ΦESI/ΦESI-like-positive isolates (≥73 ΦESI CDSs) showed resistance against ΦESI, while those lacking ΦESI CDSs were susceptible to ΦESI-mediated lysis. Of note, isolates FA1662, FA1547, and FA0720 harbored 23 CDSs only, but still showed lysis resistance. Considering the presence/absence pattern of the ΦESI CDSs in the tested isolates and their susceptibility, CDSs 006–012, 017, 027, 033–035, 038, 042, 043, 080, and 081 are candidates to be involved in a superinfection exclusion mechanism that protects against ΦESI-mediated lysis. However, most of these CDSs are predicted to encode hypothetical proteins, structural phage proteins, or products with no obvious immunity function. Moreover, although the *in silico* analysis of anti-phage systems using PADLOC v2.0 (45) did not reveal a unique profile correlating with the EOP results, we cannot completely rule out the role of particular defense systems in the lysis-resistance phenotype, especially in isolates harboring 23 ΦESI CDSs (see Table S9 at https://doi.org/10.6084/m9.figshare.31821361). Nevertheless, as 13 out of the 14 genomes showing lysis resistance harbored 23 or more ΦESI CDSs, we consider these prophage genes as the major contributors to the observed phenotype. Since the bioinformatic and phylogenomic analysis of the *Salmonella* Infantis genomes at a global scale revealed the presence of ΦESI/ΦESI-like prophages almost exclusively within the emergent *Salmonella* Infantis lineage, our results strongly suggest that the interaction observed between the PM57 and DR006 strains is not limited to these two isolates but rather represents a general phenomenon. These findings point to ΦESI/ΦESI-like prophages as promoting a competitive dominance over non-lysogenic *Salmonella* Infantis, thereby contributing, together with the pESI megaplasmid, to the successful dissemination of emergent *Salmonella* Infantis across the world.

The widespread presence of ΦESI/ΦESI-like prophages within the emergent *Salmonella* Infantis lineage may have influenced the host range of the conjugative pESI/pESI-like megaplasmids. Prophages can substantially limit the spread of conjugative plasmids by killing susceptible transconjugant bacteria, thereby shrinking the pool of viable plasmid recipients (46). The current evidence shows that the pESI megaplasmids are restricted to the emergent lineage, in which they spread mostly by clonal expansion, and no other *Salmonella* Infantis lineages carry this genetic element (7, 47) (Fig. 3D). In contrast, several different *Salmonella* serovars have acquired pESI, evidencing its transfer to and maintenance within non-Infantis lineages (47–50). Therefore, we hypothesize that the ΦESI/ΦESI-like prophages contribute to limiting the acquisition of the pESI megaplasmid by other *Salmonella* Infantis outside the emergent lineage. At the same time, pESI-like megaplasmids may also have actively contributed to the stabilization of ΦESI/ΦESI-like prophages within emergent *Salmonella* Infantis. The CDS 021 of ΦESI is predicted to encode a LexA-family protein of 250 amino acids with 32.8% amino acid identity to the cI repressor of the Lambda phage, containing a Lambda repressor-like DNA-binding domain from positions 3 to 95, and a LexA/Signal peptidase domain from positions 138 to 232 (see Fig. S8 at https://doi.org/10.6084/m9.figshare.31821361). During the SOS response, the activated RecA protein (RecA*) cleaves the cI repressor between the Ala_111_-Gly_112_ residues, allowing the transcription of the Lambda lytic-associated genes (51). These residues are also conserved in the CDS 021 (see Fig. S8 at https://doi.org/10.6084/m9.figshare.31821361), suggesting that induction of the ΦESI lytic cycle could be triggered by the host SOS response. The pESI/pESI-like megaplasmids harbor a homolog (locus tag APL92_RS24015 in pN55391) of the *psiB* gene that encodes the RecA-binding protein with anti-SOS-response activity, thereby protecting the host from lysis by preventing inadvertent prophage induction during stressful conditions (51–53). Additionally, the loss of this plasmid-encoded safeguard can leave hosts more vulnerable to SOS-driven prophage activation (54). Together, these mechanisms could have favored the coexistence of ΦESI/ΦESI-like prophages and pESI/pESI-like megaplasmids within emergent *Salmonella* Infantis, shaping the successful spread of this pathogen.

Bacterial chromosomes can undergo large inversions, commonly via homologous recombination between repeated DNA sequences and mobile elements, including prophages, which are frequent mediators or markers of chromosomal rearrangements (55). ΦESI is integrated at the 3′-end of an Arg-tRNA located approximately at 1.45 Mbp in strain PM57. However, we also detected ΦESI genes at position ≈3.05 Mbp, separated by an intermediate region of ≈1.5 Mbp (Fig. 2A). We observed the same overall pattern across 105 out of 146 closed *Salmonella* Infantis chromosomes, displaying four different arrangements based on the location of ΦESI at either the ≈1.45 or ≈3.05 Mbp loci, and the surrounding genetic context. We detected that in 39.0% of the chromosomes, a large-scale inversion of the ≈1.5 Mbp intermediate region was present, dramatically changing the genome synteny (Fig. 4). While DNA rearrangements may confer adaptive advantages resulting from gene-dosage effects derived from proximity to the replication origin (55, 56), the large-scale inversions that we detected in emergent *Salmonella* Infantis likely do not perturb transcriptional balance or mean gene copy number, as they are almost symmetrical to the *oriC*, thereby preserving gene distances.

Our study is not free of limitations, and additional experiments would strengthen the evidence for a causal role of ΦESI in the competitive advantage over ΦESI-negative strains. One limitation is that we could not carry out direct quantification of bacterial loads in the PM57-DR006 competition assays because DR006 is antibiotic susceptible and therefore could not be selectively enumerated; instead, its abundance had to be inferred by subtracting the PM57 counts from the total bacterial population. To overcome this limitation, we designed a strain-specific qPCR assay. However, the PM57-specific qPCR target lies within a putative prophage region that constitutes the only genomic difference between DR006 and PM57, apart from the ΦESI-related sequences and the conjugative pESI-like megaplasmid. Nevertheless, phage isolation from a second independent DR006–PM57 coculture again resulted in the recovery of ΦESI, as confirmed by WGS, supporting the conclusion that ΦESI is the predominant phage amplified under the coculture conditions and that any contribution of the second prophage is likely minimal. Construction of a marked PM57 strain lacking ΦESI would allow direct assessment of the specific contribution of ΦESI in competition assays using an otherwise isogenic background. Likewise, curing the pESI-like megaplasmid from PM57 would enable direct evaluation of whether plasmid carriage influences ΦESI infection dynamics, lysogenization frequency, phage production, and the competitive phenotype observed during coculture. Finally, although our results indicate that ΦESI is constitutively induced at low basal levels (Fig. 4A), we did not investigate the potential role of external factors (e.g., temperature, antibiotic at subinhibitory concentrations, etc.) as prophage inducers, which may increase the release of infective particles under biologically relevant conditions. Investigating these factors would provide further insight into the ecological role of ΦESI and its contribution to the emergence and successful dissemination of the emergent *Salmonella* Infantis lineage.

### Conclusions

Pathogen emergence is a complex, multifactorial process in which horizontal gene transfer plays a critical role (57). Our findings provide strong evidence that ΦESI and ΦESI-like prophages are active contributors to the ecological and adaptive dynamics of the emergent *Salmonella* Infantis lineage by functioning as competitive agents that selectively eliminate susceptible competitors and potentially shape the distribution of the pESI/pESI-like megaplasmids. The coexistence of both mobile elements within emergent *Salmonella* Infantis likely involves still-unexplored interactions that may have contributed to the stabilization, persistence, and global dissemination of this successful lineage.

## MATERIALS AND METHODS

### Bacterial strains and growth conditions

Twenty-one *Salmonella* Infantis isolates isolated from surface waters (*n* = 19) (18), poultry meat (*n* = 1) (15), and poultry raised in backyard production systems (*n* = 1) (58) were used for experiments in this work. Detailed information on these isolates is provided in Table S8 at https://doi.org/10.6084/m9.figshare.31821361 of the supplementary material. *Salmonella* Infantis was cultured in trypticase soy broth (TSB) or trypticase soy agar (TSA; agar 1.5% [wt/vol]) at 37°C. Tetracycline (20 µg/mL) was added to TSA plates when required.

### Phage isolation and purification

Phage ΦESI was isolated from a coculture of the *Salmonella* Infantis strains DR006 and PM57. One hundred milliliters of TSB was inoculated with 500 µL of the overnight culture of each *Salmonella* Infantis strain and incubated at 37°C overnight without shaking. One milliliter of the coculture was filtered through a 0.22 µm pore-size syringe filter. Then, 10 µL of the filtrate was spotted on a double-layer agar of the host (PM57 or DR006). After overnight incubation at 37°C, the presence or absence of lysis was observed. Plaques were further purified by four passages. Lysis (for the first passage) or single plaques (for the second and third passages) were purified by picking with a micropipette tip into the plaque, followed by resuspending the phages in SM buffer (100 mM NaCl, 8 mM MgSO_4_, 50 mM Tris-HCl, pH 7.5). A single plaque of the fourth passage was picked and used for phage amplification and preparation of a concentrated phage solution (lysate) by precipitation using PEG8000 (Polyethylene Glycol 8000) for further experiments.

### Transmission electron microscopy (TEM)

Morphotype of ΦESI was determined by TEM. Twelve microliters of the concentrated phage solution (10^10^ PFU/mL) were stained with 2% uranyl acetate and then added to a copper TEM grid. The grid with the stained sample was observed using a transmission electron microscope at the Advanced Microscopy Unit at Pontificia Universidad Católica de Chile (Talos F200X G2 TEM, Detection Ceta 16M CMOS Camera, pixel size 14 × 14 µm, 16 bit; Electron source: X-FEG Field Emission Gun). The images were analyzed using Fiji ImageJ (version: 2.14.0/1.54f).

### DNA extractions and purification

Phage genomic DNA (gDNA) was extracted from a concentrated phage lysate (10¹⁰ PFU/mL) using phenol–chloroform extraction followed by ethanol precipitation, as previously described (59), with minor modifications. Briefly, 1 mL of concentrated phage suspension was treated with 5 µL of DNase I (1 µg/mL), 6 µL of RNase A (30 µg/mL), and 10 µL of CaCl_2_ (100 mM). This mix was incubated at room temperature for 30 min to remove contaminating host nucleic acids. DNase I activity was subsequently inactivated by the addition of 40 µL of EDTA (0.5 M). Protein digestion was performed by adding 6 µL of proteinase K (20 mg/mL) and 30 µL of SDS (20%, [wt/vol]), then incubating at 56°C for 1 h. After cooling to room temperature, an equal volume (1 mL) of phenol was added, mixed gently by inversion, and centrifuged at 2,356*g* (5,000 rpm) for 5 min. The aqueous phase was carefully transferred to a new tube and extracted with an equal volume of phenol:chloroform (1:1), then centrifuged under the same conditions. The aqueous phase was transferred to a fresh tube and further extracted with an equal volume of chloroform, then centrifuged as described above. DNA was precipitated by adding 0.1 volumes of sodium acetate (3 M), followed by the addition of two volumes of 100% ethanol. Samples were gently mixed and incubated overnight at 4°C. DNA was pelleted by centrifugation at 14,000*g* (12,000 rpm) for 15 min at 4°C. The supernatant was discarded, and the pellet was washed with 100% ethanol, followed by centrifugation under the same conditions. After carefully removing the supernatant, the pellet was air-dried at room temperature until residual ethanol had completely evaporated. Finally, the DNA was resuspended in 50 µL of nuclease-free water.

gDNA of *Salmonella* Infantis PM57 was purified from 3 mL of an overnight culture grown in TSB at 37°C. The bacterial culture was pelleted by centrifugation at 8,000*g* (9,069 RPM) for 7 min at 4°C and then resuspended in 180 μL of buffer ATL from the QIAGEN DNeasy Blood and Tissue kit. Twenty microliters of Proteinase K (600 mAU/mL) were added and mixed by vortexing. The suspension was incubated at 56°C for 30 min for cell lysis. Total DNA was extracted using phenol:chloroform:isoamyl alcohol, precipitated with 3 M sodium acetate (pH 5.2), and 100% propan-2-ol, washed with 75% ethanol, and resuspended in 50 μL of nuclease-free water, as previously described (60). Genomic DNA from the competition assays was extracted using the same procedure outlined for PM57.

The concentration and purity of the obtained phage and bacterial DNA were assessed using a Nanodrop spectrophotometer or a Qubit 3.0 fluorometer. The DNA was stored at −20°C until required.

### WGS, assembly, and annotation

Bacteriophage genomic DNA was sequenced using Illumina technology. DNA samples were submitted to MicrobesNG (Birmingham, UK) for library preparation and sequencing. Genomic libraries were prepared using the Nextera XT Library Prep Kit, following the manufacturer’s protocol with minor modifications: the input DNA amount was increased twofold, and the PCR elongation time was extended to 45 s to improve library complexity. DNA quantification and library preparation were performed using a Hamilton Microlab STAR automated liquid handling system. Libraries were sequenced using a 250 bp paired-end protocol. Raw reads were quality-filtered and adapter-trimmed using Trimmomatic v0.30 (61) with a sliding-window quality cutoff of Q15. *De novo* genome assembly was performed using SPAdes v3.7 (62). Contigs were annotated using Pharokka v1.7.5 with the v1.4.0 database (63).

Purified gDNA of *Salmonella* Infantis PM57 was sent to SeqCenter LLC (Pittsburgh, USA) for hybrid WGS using Illumina and Oxford Nanopore Technologies (ONT) platforms, and genome assembly. Illumina sequencing libraries were prepared using the tagmentation-based and PCR-based Illumina DNA Prep kit and custom IDT 10 bp unique dual indices with a target insert size of 280 bp. No additional DNA fragmentation or size selection steps were performed. Illumina sequencing was performed on an Illumina NovaSeq X Plus sequencer in one or more multiplexed shared-flow-cell runs, producing 2 × 151 bp paired-end reads. Demultiplexing, quality control, and adapter trimming were performed with bcl-convert (v4.2.4). Long-read libraries were prepared using the PCR-free ONT Ligation Sequencing Kit (SQK-NBD114.96) with the NEBNext Companion Module (E7180L) according to the manufacturer’s specifications. No additional DNA fragmentation or size selection was performed. Nanopore sequencing was performed on an ONT GridION or PromethION sequencer using R10.4.1 flow cells in one or more multiplexed shared-flow-cell runs. The run design used the 400 bps sequencing mode with a minimum read length of 200 bp. The Dorado v0.5.3 basecaller (https://github.com/nanoporetech/dorado) was used under the super-accurate (sup) and 5mC/5hmC (5mC_5hmC) basecalling models. Dorado demultiplex was used under the SQK-NBD114.96 kit settings to generate the BAM file. SAMtools fastq v1.20 (64) was used to generate the FASTQ file from the BAM file.

Porechop v0.2.4 (https://github.com/rrwick/Porechop) was used with default parameters to trim residual adapter sequences from the ONT reads that may have been missed during basecalling and demultiplexing. *De novo* genome assemblies were generated from the ONT read data with Flye v2.9.2 (65) under the nano-hq (ONT high-quality reads) model. Disjointing assemblies with the longest reads that provided 50× coverage (--asm-coverage), assuming a genome size of 6 megabases (--genome-size), were included. Subsequent polishing used the Illumina read data with Pilon v1.24 (66) under default parameters. To reduce erroneous assembly artifacts caused by low-quality nanopore reads, long-read contigs with an average short read coverage of 15× or less were removed from the assembly. Assembled contigs were evaluated for circularization via Circlator v1.5.5 (67) using the ONT long reads. Assembly annotation was performed with Bakta v1.10.1 using the Bakta v5.1 database (68).

### Taxonomic analysis

To assess the taxonomy of ΦESI, a BLASTn search against the NCBI Viral Nucleotide Collection in NCBI Virus (https://www.ncbi.nlm.nih.gov/labs/virus/vssi/#/) was performed using the ΦESI assembly as query. The genomes from the top 8 BLASTn results were downloaded and used to carry out taxonomic analyses using the VICTOR and VIRIDIC web platforms, using default parameters and the d6 equation for VICTOR (69, 70).

### Distribution analysis of ΦESI genes among global *Salmonella* Infantis genomes

Metadata, assembly statistics, and cgMLST-based hierarchical cluster information were retrieved for all 36,499 *Salmonella* Infantis genomes publicly available in EnteroBase (71) as of 9 September 2025. Low-quality and redundant genomes were filtered out as previously described (7). Briefly, assemblies with N50 < 100,000 and coverage <30× were removed from the data set. Redundant genomes with the same collection year, source niche, country of origin, and from the same HC5 cluster (linked by ≤5 cgMLST allele differences) were removed, leaving only one representative genome in order to retain most of the temporal, geographic, isolation source, and genomic diversity. After this process, 20,429 non-redundant genomes were selected and downloaded for further analysis (see Table S3 at https://doi.org/10.6084/m9.figshare.31821361).

The presence of the ΦESI genes in the non-redundant *Salmonella* Infantis data set was assessed using ABRicate v1.0.1 (https://github.com/tseemann/abricate) and a custom database made with the 88 ΦESI CDSs identified with Pharokka (see Table S4 at https://doi.org/10.6084/m9.figshare.31821361). Then, a non-clonal subset of 15,707 genomes (>5 cgMLST-allele differences) was selected to assess the ΦESI CDS-number distribution, their similarity [(%identity×%coverage)/100], and their synteny relative to the ΦESI virion genome to identify major *Salmonella* Infantis groups. The presence of pESI-like megaplasmids was also assessed with ABRicate and a custom database as previously described (7).

A recombination-free core-SNP maximum likelihood phylogenomic tree was constructed based on 864 non-redundant *Salmonella* Infantis genomes (according to collection year, country, and HC50 information) from the cluster HC200_36, which encompasses all genomes positive for ΦESI and ΦESI-like prophages. This phylogeny was constructed as recently described (7). Briefly, the whole-genome alignment was obtained with Snippy v.4.6.0 (https://github.com/tseemann/snippy) using the contigs of the 864 genomes and the closed chromosome of *Salmonella* Infantis strain N55391 as the reference (EnteroBase assembly barcode SAL_XA0374AA_AS), using default parameters. Recombination regions were detected with Gubbins v3.3 (72) after five iterations using the iqtree-fast option and the Jukes-Cantor model for the first iteration, and the –best-model option for the remaining iterations. The whole-genome alignment with masked recombination regions obtained from Gubbins was used as the input to snp-sites v2.5.1 (73) to obtain the recombination-free core-SNP alignment. The core-SNP phylogenomic tree was constructed with IQ-Tree MPI multicore v2.3.2 (74) using the GTR + G4 + F + ASC nucleotide substitution model, which was selected in the Gubbins iterations. Node support was assessed with 1,000 ultrafast bootstraps (75). The resulting tree was uploaded to iTOL v7 (76) and rooted to the best-fitting temporal root estimated with TempEst v1.5.3 (77). The geographic information (regions of origin; geoscheme of the United Nations), megaplasmid presence, and number of ΦESI CDSs were integrated into the phylogeny.

### Analysis of prophage ΦESI genomic context

A new custom ABRicate database, containing one sequence corresponding to the complete ΦESI genome, was used to screen all available *Salmonella* Infantis genomes retrieved from NCBI Pathogen Detection (22 May 2025); only genomes designated as “Complete” in Pathogen Detection were included in the screening (see Table S4 at https://doi.org/10.6084/m9.figshare.31821361). ABRicate summary outputs were used to define presence/absence matrices and to identify distinct prophage insertion profiles across the data set.

For each identified insertion site, the genomic context was defined by extracting 10 kb upstream and 10 kb downstream of the insertion coordinates from representative genomes. Flanking sequences were clustered with cd-hit-est v4.8.1 (https://github.com/weizhongli/cdhit) at 99% nucleotide identity to reduce redundancy and to group highly similar genomic contexts; a single representative sequence from each cluster was selected for downstream visualization and comparative interpretation. Custom scripts were used to parse ABRicate outputs, extract flanking regions, perform clustering, and select representative genomes/contexts. These scripts are available in the project repository (see Data Availability). Finally, representative genomes for each unique insertion profile (and separately for genomes exhibiting large-scale structural rearrangements) were chosen for whole-genome structural comparison and visualization using pyGenomeViz (https://github.com/moshi4/pyGenomeViz). All bioinformatic analyses were performed on a Linux computer server using 12 CPU threads unless otherwise stated; default tool parameters were used unless specified above.

### Competition assay between emergent and non-emergent *Salmonella* Infantis

A competition assay was performed between *Salmonella* Infantis strain PM57 (emergent clade) and strain DR006 (non-emergent). Individual, overnight cultures of the two strains were prepared in TSB at 37°C. Both strains were cocultured in 100 mL TSB at an initial concentration of 10⁶ CFU/mL (1:1 ratio) and incubated at 37°C and 175 rpm. Samples were collected hourly for 6 h and again after 24 h to quantify phage concentration (PFU/mL) in a double-agar overlay and bacterial load by colony counting (CFU/mL).

To estimate phage concentration, aliquots (<1 mL) from the cocultures were filtered through a 0.22 µm pore-size syringe filter. Subsequently, 20 µL of the filtrate was serially diluted tenfold (up to 10^−8^) in SM buffer. Then, 5 µL of each dilution was spotted in triplicate onto double-layer agar plates containing *Salmonella* Infantis DR006 as the host strain. Then, the plates were incubated overnight at 37°C. Plaque-forming units were counted to determine the concentration expressed in PFU per milliliter. All assays were performed independently three times (three biological replicates).

To estimate the total bacterial loads (PM57 + DR006), 20 µL of each sample was serially diluted tenfold (up to 10⁻⁸) in 0.9% NaCl. Then, 10 µL of each dilution was spotted in triplicate onto TSA plates and incubated at 37°C overnight. To specifically quantify strain PM57, 10 µL of each dilution was also spotted in triplicate onto TSA supplemented with tetracycline (20 µg/mL), as PM57 is resistant to tetracycline (IC_50_ = 163.9 µg/mL), while DR006 is susceptible (IC_50_ = 1.5 µg/mL; see Fig. S9 at https://doi.org/10.6084/m9.figshare.31821361). Plates were incubated overnight at 37°C.

The concentration of strain DR006 was calculated indirectly by subtracting the obtained value for PM57 from the total bacterial load according to the following formula:


$$DR006_{\frac{CFU}{mL}}=Total\;Bacteria_{\frac{CFU}{mL}}-PM57_{\frac{CFU}{mL}}.$$

As controls, each strain was also cultured individually (monoculture) under the same conditions, and bacterial concentrations were determined at the same time points.

CI of PM57 over DR006 in the coculture at each time point was calculated according to the following formula:


$$CI=\frac{{\left(PM57_{\frac{CFU}{mL}}/DR006_{\frac{CFU}{mL}}\right)}_{t_{x}}}{{\left(PM57_{\frac{CFU}{mL}}/DR006_{\frac{CFU}{mL}}\right)}_{t_{0}}}.$$

The denominator of the formula represents the average $PM57_{\frac{CFU}{mL}}/DR006_{\frac{CFU}{mL}}$ ratio at time 0, calculated from three independent biological replicates.

### qPCR quantification

Targeted qPCR assays were performed to distinguish between PM57 and DR006 in the competition assays and to estimate their absolute loads (see Fig. S10 and S11 at https://doi.org/10.6084/m9.figshare.31821361). For PM57 quantification, qPCR reactions were conducted using 50 ng of template DNA per reaction and primers targeting the baseplate X gene, which is present in the PM57 chromosome but absent from the DR006 chromosome (see Fig. S10 at https://doi.org/10.6084/m9.figshare.31821361). This gene is part of a putative prophage, which represents the only differing region between both strains besides the pESI megaplasmid and the ΦESI-related sequences. The following primers were used: PM57_qPCR_Fw 5′-CTGAACGTCGGGCAGTT-3′ and PM57_qPCR_Rev 5′-CTGAGGGCGTGGTTGAG-3′, which produce an amplicon of 101 bp in strain PM57 but not in DR006. For DR006 detection, qPCR reactions were carried out using 50 ng of template DNA per reaction and primers targeting a genomic region spanning the interval between the *fieF* and *cpxP* genes. In the PM57 chromosome, these primers are separated by 31,676 bp (see Fig. S10 at https://doi.org/10.6084/m9.figshare.31821361); therefore, no amplification is expected in PM57 under standard qPCR conditions, which cannot amplify such a large genomic fragment, ensuring assay specificity for DR006. The primers used were DR006_qPCR_Fw 5′-GCTAATAACAGAATGACAAACTGGAG-3′ and DR006_qPCR_Rev 5′-CAACTCACGTTCCCAGTAACA-3′, producing an amplicon of 113 bp in DR006.

All qPCR reactions were performed with the PowerUp SYBR Green Master Mix (Applied Biosystems) in a QuantStudio 5 real-time PCR system with the QuantStudio Design and Analysis Software v1.5.2, following the master mix specifications.

### Isolation of DR006-derived ΦESI-lysis-resistant mutants and WGS analysis

An overnight culture of strain DR006 was diluted in TSB to 10^5^ CFU/mL. A ΦESI stock was similarly diluted in SM buffer to 2 × 10^7^ PFU/mL. Fifty microliters of the diluted bacterial culture were mixed with 50 µL of ΦESI in a 96-well microplate (final volume 100 µL, multiplicity of infection = 200) and incubated overnight at 37°C, alongside an uninfected control culture. Following incubation, an aliquot of the infected culture was streaked onto TSA plates to isolate surviving bacteria, presumptive phage-resistant mutants. A single colony was selected and subjected to three serial passages on TSA, with a single colony isolated and re-streaked at each passage to eliminate infective phage particles from the original culture and to ensure clonal purity. The parental wild-type DR006 strain was subcultured in parallel under identical conditions. After each passage, resistance to the ΦESI-mediated lysis was assessed by efficiency-of-plating assays using soft-agar overlays challenged with serial phage dilutions, comparing the candidate mutant to wild-type DR006. Two independent DR006-derived mutants were obtained, which consistently displayed resistance to ΦESI-mediated lysis. These strains were named R4 and 3φ. The subcultured DR006 control was named W1. These strains were cryopreserved in 30% glycerol at −80°C.

Whole-genome sequencing of the W1, R4, and 3φ strains was carried out on SeqCenter, using genomic DNA extracted as described in the above sections. The raw reads were uploaded to the Sequence Read Archive (see Data Availability section). *De novo* genome assembly of the trimmed, quality-filtered reads was performed with Unicycler v0.5.1 (78), with a minimum contig length of 500 bp and retention of intermediate files. Assembly quality was evaluated using QUAST v5.3.0 (79), and completeness/contamination was assessed with CheckM2 v1.1.0 (80). Assemblies were annotated with Bakta v1.8.1 using the corresponding database under default settings. To identify nucleotide variants distinguishing R4 and 3φ from W1, the reads from each strain were mapped against the assembled W1 genome using Snippy v4.6.0 with default parameters. Variants common to 3φ and R4, relative to the W1 wild-type reference, were identified from the resulting core/variant calls. Whole-genome comparisons using Mauve v20150226 (81) were also carried out to assess the presence of indels spanning several nucleotides and rearrangements. Finally, the presence of ΦESI and ΦESI-related sequences in the R4, 3φ, and W1 genomes was assessed with PhiSpy v4.2.21 (82) for prophage prediction and ABRicate v1.0.1 with the custom ΦESI database, as described in the sections above.

### Susceptibility assays on *Salmonella* Infantis strains carrying ΦESI and ΦESI-like prophages

The susceptibility to lysis mediated by ΦESI viral particles of different *Salmonella* Infantis representing pESI+/ΦESI+, pESI−/ΦESI-like+, and pESI−/ΦESI-negative isolates (see Table S1 at https://doi.org/10.6084/m9.figshare.31821361) was determined using the EOP assay. A suspension of phage ΦESI was serially diluted from 10^−1^ to 10^−8^ in SM buffer, and 5 µL of each dilution was spotted on a soft TSA agar overlay (0.7% agar [wt/vol]) seeded with each *Salmonella* isolate. The plates were incubated overnight at 37°C. The efficiency of plating was calculated by dividing the resulting phage titer of the tested isolate by the titer of the reference isolate *Salmonella* Infantis DR006.

### Statistical analysis

Statistical analyses were performed to compare growth dynamics and competitive performance between bacterial strains (PM57 and DR006 in competition). Growth curves were analyzed by calculating the AUC. Differences in AUC between monocultures and cocultures for each strain were assessed using a two-way ANOVA followed by multiple comparison tests. For AUC values obtained from qPCR-based quantification, comparisons were performed using Welch’s *t*-test to account for unequal variances between strains in coculture. Competition index values across time points were analyzed using an ordinary one-way ANOVA followed by Dunnett’s multiple comparisons test, where all time points were compared against time 0 as the control condition. Statistical significance was defined according to the indicated *P values* for each statistical analysis. All statistical analyses were performed using GraphPad Prism Version 10.2.3.

The association between the presence of ΦESI/ΦESI-like prophages and the global emergent *Salmonella* Infantis lineage (represented by carriage of pESI-like megaplasmids) (7) was assessed with a *χ*^2^ test. The 95% CI was computed on the log scale using the Wald approximation. The geographic distribution of insertion profiles of ΦESI/ΦESI-like prophages among *Salmonella* Infantis isolates with an available complete genome was assessed through two-sided Fisher’s exact tests. All tests were performed in GraphPad Prism Version 10.2.3, with α = 0.05.

## Supplementary Material

Reviewer comments

## Data Availability

All data are available in this manuscript and its Supplemental Materials. Supplemental Figures and Tables are available in this link: https://doi.org/10.6084/m9.figshare.31821361. Genome assemblies of ΦESI and PM57 are available at NCBI under accession numbers PZ014354.1 and JBUZKO000000000.1, respectively. Sequencing reads from wild-type DR006 and derived lysis-resistant mutants R4 and 3phi are available under BioProject accession PRJNA1494598. All custom scripts used in this study are available at the GitHub repository BaFoP-Lab/phiESI_2026_code_repo.

## SUPPLEMENTAL MATERIAL

OPEN PEER REVIEW reviewer-comments.pdf An accounting of the reviewer comments and feedback.

ASM does not own the copyrights to Supplemental Material that may be linked to, or accessed through, an article. The authors have granted ASM a non-exclusive, world-wide license to publish the Supplemental Material files. Please contact the corresponding author directly for reuse.

## References

[1] GBD 2019 Antimicrobial Resistance Collaborators. 2022. Global mortality associated with 33 bacterial pathogens in 2019: a systematic analysis for the global burden of disease study 2019. Lancet 400:2221–2248. doi:10.1016/S0140-6736(22)02185-7 PMC9763654 36423648

[2] CDC. 2024. Salmonella infection - clinical overview of salmonellosis. Available from: https://www.cdc.gov/salmonella/hcp/clinical-overview/index.html

[3] Katz TS, Harhay DM, Schmidt JW, Wheeler TL. 2024. Identifying a list of *Salmonella* serotypes of concern to target for reducing risk of salmonellosis. Front Microbiol 15:1307563. doi:10.3389/fmicb.2024.1307563 PMC10894960 38410382

[4] Piña-Iturbe A, Adell AD, Reyes-Jara A, Toro M, Moreno-Switt AI. 2025. *Salmonella* in Chile: an urgent need for timely data and WGS implementation. The Lancet Regional Health - Americas 48:101179. doi:10.1016/j.lana.2025.101179 PMC12343471 40809294

[5] European Food Safety Authority (EFSA), European Centre for Disease Prevention and Control (ECDC). 2025. The European Union One Health 2024 Zoonoses Report. EFSA J 23:e9759. doi:10.2903/j.efsa.2025.9759 PMC12686834 41377167

[6] Alvarez DM, Barrón-Montenegro R, Conejeros J, Rivera D, Undurraga EA, Moreno-Switt AI. 2023. A review of the global emergence of multidrug-resistant *Salmonella enterica* subsp. enterica Serovar Infantis. Int J Food Microbiol 403:110297. doi:10.1016/j.ijfoodmicro.2023.110297 37406596

[7] Piña-Iturbe A, Tichy-Navarro D, Miranda-Riveros J, Navarrete MJ, Moreno-Switt AI. 2026. Emergent *Salmonella enterica* serovar Infantis forms a monophyletic lineage shaped by geographic structuring. Int J Food Microbiol 446:111536. doi:10.1016/j.ijfoodmicro.2025.111536 41330089

[8] Aviv G, Tsyba K, Steck N, Salmon-Divon M, Cornelius A, Rahav G, Grassl GA, Gal-Mor O. 2014. A unique megaplasmid contributes to stress tolerance and pathogenicity of an emergent *Salmonella enterica* serovar Infantis strain. Environ Microbiol 16:977–994. doi:10.1111/1462-2920.12351 24320043

[9] Aviv G, Elpers L, Mikhlin S, Cohen H, Vitman Zilber S, Grassl GA, Rahav G, Hensel M, Gal-Mor O. 2017. The plasmid-encoded Ipf and Klf fimbriae display different expression and varying roles in the virulence of *Salmonella enterica* serovar Infantis in mouse vs. avian hosts. PLoS Pathog 13:e1006559. doi:10.1371/journal.ppat.1006559 PMC5560535 28817673

[10] Gal-Mor O, Valinsky L, Weinberger M, Guy S, Jaffe J, Schorr YI, Raisfeld A, Agmon V, Nissan I. 2010. Multidrug-resistant *Salmonella enterica* serovar Infantis, Israel. Emerg Infect Dis 16:1754–1757. doi:10.3201/eid1611.100100 PMC3294505 21029536

[11] Aviv G, Rahav G, Gal-Mor O. 2016. Horizontal transfer of the *Salmonella enterica* serovar Infantis resistance and virulence plasmid pESI to the gut microbiota of warm-blooded hosts. mBio 7:e01395-16. doi:10.1128/mBio.01395-16 PMC5013300 27601577

[12] Tate H, Folster JP, Hsu C-H, Chen J, Hoffmann M, Li C, Morales C, Tyson GH, Mukherjee S, Brown AC, Green A, Wilson W, Dessai U, Abbott J, Joseph L, Haro J, Ayers S, McDermott PF, Zhao S. 2017. Comparative analysis of extended-spectrum-β-lactamase CTX-M-65-producing *Salmonella enterica* serovar Infantis isolates from humans, food animals, and retail chickens in the United States. Antimicrob Agents Chemother 61:e00488–17. doi:10.1128/AAC.00488-17 PMC5487606 28483962

[13] Alba P, Leekitcharoenphon P, Carfora V, Amoruso R, Cordaro G, Di Matteo P, Ianzano A, Iurescia M, Diaconu EL, ENGAGE-EURL-AR Network Study Group, Pedersen SK, Guerra B, Hendriksen RS, Franco A, Battisti A. 2020. Molecular epidemiology of *Salmonella* Infantis in Europe: insights into the success of the bacterial host and its parasitic pESI-like megaplasmid. Microb Genom 6:e000365. doi:10.1099/mgen.0.000365 PMC7371121 32271142

[14] Drauch V, Kornschober C, Palmieri N, Hess M, Hess C. 2021. Infection dynamics of *Salmonella* Infantis strains displaying different genetic backgrounds - with or without pESI-like plasmid - vary considerably. Emerg Microbes Infect 10:1471–1480. doi:10.1080/22221751.2021.1951124 PMC8300933 34197273

[15] Piña-Iturbe A, Díaz-Gavidia C, Álvarez FP, Barron-Montenegro R, Álvarez-Espejo DM, García P, Solís D, Constenla-Albornoz R, Toro M, Olivares-Pacheco J, Reyes-Jara A, Meng J, Bell RL, Moreno-Switt AI. 2024. Genomic characterisation of the population structure and antibiotic resistance of *Salmonella enterica* serovar Infantis in Chile, 2009-2022. Lancet Reg Health Am 32:100711. doi:10.1016/j.lana.2024.100711 PMC10944094 38495315

[16] Gebreyes WA, Wittum T, Habing G, Alali W, Usui M, Suzuki S. 2017. Spread of antibiotic resistance in food animal production systems. *In* Foodborne diseases: third edition. Elsevier Inc.

[17] Srednik ME, Morningstar-Shaw BR, Hicks JA, Tong C, Mackie TA, Schlater LK. 2023. Whole-genome sequencing and phylogenetic analysis capture the emergence of a multi-drug resistant *Salmonella enterica* serovar Infantis clone from diagnostic animal samples in the United States. Front Microbiol 14:1166908. doi:10.3389/fmicb.2023.1166908 PMC10272548 37333652

[18] Chen Z, Moreno-Switt AI, Reyes-Jara A, Delgado Suarez E, Adell AD, Oliveira CJB, Bonelli RR, Huang X, Brown E, Allard M, Grim C, Bell R, Meng J, Toro M. 2024. A multicenter genomic epidemiological investigation in Brazil, Chile, and Mexico reveals the diversity and persistence of *Salmonella* populations in surface waters. mBio 15:e00777-24. doi:10.1128/mbio.00777-24 PMC11253603 38920393

[19] Fuentes-Castillo D, Farfán-López M, Esposito F, Moura Q, Fernandes MR, Lopes R, Cardoso B, Muñoz ME, Cerdeira L, Najle I, Muñoz PM, Catão-Dias JL, González-Acuña D, Lincopan N. 2019. Wild owls colonized by international clones of extended-spectrum β-lactamase (CTX-M)-producing *Escherichia coli* and *Salmonella* Infantis in the Southern Cone of America. Sci Total Environ 674:554–562. doi:10.1016/j.scitotenv.2019.04.149 31022545

[20] Kim Y, Cho H, Lee M, Jung S-H, Chung Y-J, Ko KS, Park KT. 2025. Molecular epidemiology of pESI-carrying *Salmonella* Infantis in Korea: insights from a one health framework. Emerg Microbes Infect 14:2565389. doi:10.1080/22221751.2025.2565389 PMC12507103 40977415

[21] Dion MB, Oechslin F, Moineau S. 2020. Phage diversity, genomics and phylogeny. Nat Rev Microbiol 18:125–138. doi:10.1038/s41579-019-0311-5 32015529

[22] Salmond GPC, Fineran PC. 2015. A century of the phage: past, present and future. Nat Rev Microbiol 13:777–786. doi:10.1038/nrmicro3564 26548913

[23] Howard-Varona C, Hargreaves KR, Abedon ST, Sullivan MB. 2017. Lysogeny in nature: mechanisms, impact and ecology of temperate phages. ISME J 11:1511–1520. doi:10.1038/ismej.2017.16 PMC5520141 28291233

[24] Makky S, Dawoud A, Safwat A, Abdelsattar AS, Rezk N, El-Shibiny A. 2021. The bacteriophage decides own tracks: when they are with or against the bacteria. Curr Res Microb Sci 2:100050. doi:10.1016/j.crmicr.2021.100050 PMC8610337 34841341

[25] Landy A. 2015. The λ integrase site-specific recombination pathway. Microbiol Spectr 3:MDNA3-0051-2014. doi:10.1128/microbiolspec.MDNA3-0051-2014 PMC5710010 26104711

[26] Ghosh D, Roy K, Williamson KE, Srinivasiah S, Wommack KE, Radosevich M. 2009. Acyl-homoserine lactones can induce virus production in lysogenic bacteria: an alternative paradigm for prophage induction. Appl Environ Microbiol 75:7142–7152. doi:10.1128/AEM.00950-09 PMC2786502 19783745

[27] Silpe JE, Bassler BL. 2019. A host-produced quorum-sensing autoinducer controls a phage lysis-lysogeny decision. Cell 176:268–280. doi:10.1016/j.cell.2018.10.059 PMC6329655 30554875

[28] Silpe JE, Duddy OP, Johnson GE, Beggs GA, Hussain FA, Forsberg KJ, Bassler BL. 2023. Small protein modules dictate prophage fates during polylysogeny. Nature 620:625–633. doi:10.1038/s41586-023-06376-y PMC10432266 37495698

[29] Bruce JB, Lion S, Buckling A, Westra ER, Gandon S. 2021. Regulation of prophage induction and lysogenization by phage communication systems. Curr Biol 31:5046–5051. doi:10.1016/j.cub.2021.08.073 PMC8612742 34562385

[30] Nanda AM, Thormann K, Frunzke J. 2015. Impact of spontaneous prophage induction on the fitness of bacterial populations and host-microbe interactions. J Bacteriol 197:410–419. doi:10.1128/JB.02230-14 PMC4285972 25404701

[31] Canchaya C, Proux C, Fournous G, Bruttin A, Brüssow H. 2003. Prophage genomics. Microbiol Mol Biol Rev 67:238–276. doi:10.1128/MMBR.67.2.238-276.2003 PMC156470 12794192

[32] Yasmin A, Kenny JG, Shankar J, Darby AC, Hall N, Edwards C, Horsburgh MJ. 2010. Comparative genomics and transduction potential of *Enterococcus faecalis* temperate bacteriophages. J Bacteriol 192:1122–1130. doi:10.1128/JB.01293-09 PMC2812964 20008075

[33] Chevallereau A, Pons BJ, van Houte S, Westra ER. 2022. Interactions between bacterial and phage communities in natural environments. Nat Rev Microbiol 20:49–62. doi:10.1038/s41579-021-00602-y 34373631

[34] Godsil M, Ritz NL, Venkatesh S, Meeske AJ. 2025. Gut phages and their interactions with bacterial and mammalian hosts. J Bacteriol 207:e00428-24. doi:10.1128/jb.00428-24 PMC11844821 39846747

[35] Gama JA, Reis AM, Domingues I, Mendes-Soares H, Matos AM, Dionisio F. 2013. Temperate bacterial viruses as double-edged swords in bacterial warfare. PLoS One 8:e59043. doi:10.1371/journal.pone.0059043 PMC3594171 23536852

[36] Duerkop BA, Clements CV, Rollins D, Rodrigues JLM, Hooper LV. 2012. A composite bacteriophage alters colonization by an intestinal commensal bacterium. Proc Natl Acad Sci USA 109:17621–17626. doi:10.1073/pnas.1206136109 PMC3491505 23045666

[37] Bossi L, Fuentes JA, Mora G, Figueroa-Bossi N. 2003. Prophage contribution to bacterial population dynamics. J Bacteriol 185:6467–6471. doi:10.1128/JB.185.21.6467-6471.2003 PMC219396 14563883

[38] Gymoese P, Kiil K, Torpdahl M, Østerlund MT, Sørensen G, Olsen JE, Nielsen EM, Litrup E. 2019. WGS based study of the population structure of *Salmonella enterica* serovar Infantis. BMC Genomics 20:870. doi:10.1186/s12864-019-6260-6 PMC6858691 31730461

[39] Cohen E, Rahav G, Gal-Mor O, Ochman H. 2020. Genome sequence of an emerging *Salmonella enterica* serovar Infantis and genomic comparison with other *S. Infantis* strains. Genome Biol Evol 12:151–159. doi:10.1093/gbe/evaa048 PMC7144548 32145019

[40] Li XY, Lachnit T, Fraune S, Bosch TCG, Traulsen A, Sieber M. 2017. Temperate phages as self-replicating weapons in bacterial competition. J R Soc Interface 14:1–7. doi:10.1098/rsif.2017.0563 PMC5746566 29263125

[41] Egido JE, Costa AR, Aparicio-Maldonado C, Haas PJ, Brouns SJJ. 2022. Mechanisms and clinical importance of bacteriophage resistance. FEMS Microbiol Rev 46:1–16. doi:10.1093/femsre/fuab048 PMC8829019 34558600

[42] Liu B, Zhang L, Zhu X, Shi C, Chen J, Liu W, He X, Shi X. 2011. PCR identification of *Salmonella* serogroups based on specific targets obtained by comparative genomics. Int J Food Microbiol 144:511–518. doi:10.1016/j.ijfoodmicro.2010.11.010 21131088

[43] Liu Bin, Knirel YA, Feng L, Perepelov AV, Senchenkova SN, Reeves PR, Wang L. 2014. Structural diversity in *Salmonella* O antigens and its genetic basis. FEMS Microbiol Rev 38:56–89. doi:10.1111/1574-6976.12034 23848592

[44] Chen Y, Wang X, Zhang Q, Zhang Z, Liu Z, Zhao X, Li L, Hu M, Lv Q, Luo Y, Xu X, Petersen B, Cai Y, Sicheritz-Pontén T, Clokie MRJ, Liu Y. 2025. ABCD-type phage cocktail targeting distinct LPS receptor sites demonstrates superior efficacy against multidrug-resistant *Salmonella*. J Hazard Mater 500:140435. doi:10.1016/j.jhazmat.2025.140435 41240830

[45] Payne LJ, Meaden S, Mestre MR, Palmer C, Toro N, Fineran PC, Jackson SA. 2022. PADLOC: a web server for the identification of antiviral defence systems in microbial genomes. Nucleic Acids Res 50:W541–W550. doi:10.1093/nar/gkac400 PMC9252829 35639517

[46] Igler C, Schwyter L, Gehrig D, Wendling CC. 2022. Conjugative plasmid transfer is limited by prophages but can be overcome by high conjugation rates. Philosophical Transactions of the Royal Society B 377:20200470. doi:10.1098/rstb.2020.0470 PMC8628080 34839704

[47] Li C, Tate H, Huang X, Hsu C-H, Harrison LB, Zhao S, Fortenberry GZ, Dessai U, McDermott PF, Strain EA. 2024. The spread of pESI-mediated extended-spectrum cephalosporin resistance in *Salmonella* serovars—Infantis, Senftenberg, and Alachua isolated from food animal sources in the United States. PLoS One 19:e0299354. doi:10.1371/journal.pone.0299354 PMC10939224 38483966

[48] Cohen E, Kriger O, Amit S, Davidovich M, Rahav G, Gal-Mor O. 2022. The emergence of a multidrug resistant *Salmonella* Muenchen in Israel is associated with horizontal acquisition of the epidemic pESI plasmid. Clin Microbiol Infect 28:1499. doi:10.1016/j.cmi.2022.05.029 35654317

[49] Dos Santos AMP, Panzenhagen P, Ferrari RG, Conte-Junior CA. 2022. Large-scale genomic analysis reveals the pESI-like megaplasmid presence in *Salmonella* Agona, Muenchen, Schwarzengrund, and Senftenberg. Food Microbiol 108:104112. doi:10.1016/j.fm.2022.104112 36088119

[50] Miranda-Riveros J, Tichy-Navarro D, Navarrete MJ, Reyes-Jara A, Toro M, Ugalde JA, Moreno-Switt AI, Piña-Iturbe A. 2026. Environmental surveillance in Chile reveals a multidrug-resistant *Salmonella* Amager from an emerging bla_CTX-M-65_/pESI-positive lineage. Int J Antimicrob Agents 67:107831. doi:10.1016/j.ijantimicag.2026.107831 42103161

[51] Gavric D, Knezevic P. 2026. Antibiotic-mediated prophage activation: mechanisms, consequences, and therapeutic implications. Int J Antimicrob Agents 67:107655. doi:10.1016/j.ijantimicag.2025.107655 41167349

[52] Bailone A, Bäckman A, Sommer S, Célérier J, Bagdasarian MM, Bagdasarian M, Devoret R. 1988. PsiB polypeptide prevents activation of RecA protein in *Escherichia coli*. Mol Gen Genet 214:389–395. doi:10.1007/BF00330471 3063944

[53] Petrova V, Chitteni-Pattu S, Drees JC, Inman RB, Cox MM. 2009. An SOS inhibitor that binds to free RecA protein: the PsiB protein. Mol Cell 36:121–130. doi:10.1016/j.molcel.2009.07.026 PMC2761248 19818715

[54] Tuttle MJ, May FS, Basso JTR, Gann ER, Xu J, Buchan A. 2022. Plasmid-mediated stabilization of prophages. mSphere:e0093021. doi:10.1128/msphere.00930-21 PMC9044938 35311569

[55] Waters EV, Tucker LA, Ahmed JK, Wain J, Langridge GC. 2022. Impact of *Salmonella* genome rearrangement on gene expression. Evol Lett 6:426–437. doi:10.1002/evl3.305 PMC9783417 36579163

[56] Martinez-Gutierrez CA, Huang Y, Bobay L-M. 2026. Prevalence and evolutionary implications of genome rearrangements in bacteria. Genome Biol Evol 18:evag002. doi:10.1093/gbe/evag002 PMC12863080 41575375

[57] Balasubramanian D, López-Pérez M, Grant TA, Ogbunugafor CB, Almagro-Moreno S. 2022. Molecular mechanisms and drivers of pathogen emergence. Trends Microbiol 30:898–911. doi:10.1016/j.tim.2022.02.003 35248462

[58] Alegria-Moran R, Rivera D, Toledo V, Moreno-Switt AI, Hamilton-West C. 2017. First detection and characterization of *Salmonella* spp. in poultry and swine raised in backyard production systems in central Chile. Epidemiol Infect 145:3180–3190. doi:10.1017/S0950268817002175 PMC9148768 28927474

[59] Moreno Switt AI, den Bakker HC, Vongkamjan K, Hoelzer K, Warnick LD, Cummings KJ, Wiedmann M. 2013. *Salmonella* bacteriophage diversity reflects host diversity on dairy farms. Food Microbiol 36:275–285. doi:10.1016/j.fm.2013.06.014 PMC3779805 24010608

[60] Piña-Iturbe A, Hoppe-Elsholz G, Fernández PA, Santiviago CA, González PA, Bueno SM. 2022. Bioinformatic and experimental characterization of SEN1998: a conserved gene carried by the enterobacteriaceae-associated ROD21-like family of genomic islands. Sci Rep 12:2435. doi:10.1038/s41598-022-06183-x PMC8844411 35165310

[61] Bolger AM, Lohse M, Usadel B. 2014. Trimmomatic: a flexible trimmer for Illumina sequence data. Bioinformatics 30:2114–2120. doi:10.1093/bioinformatics/btu170 PMC4103590 24695404

[62] Bankevich A, Nurk S, Antipov D, Gurevich AA, Dvorkin M, Kulikov AS, Lesin VM, Nikolenko SI, Pham S, Prjibelski AD, Pyshkin AV, Sirotkin AV, Vyahhi N, Tesler G, Alekseyev MA, Pevzner PA. 2012. SPAdes: a new genome assembly algorithm and its applications to single-cell sequencing. J Comput Biol 19:455–477. doi:10.1089/cmb.2012.0021 PMC3342519 22506599

[63] Bouras G, Nepal R, Houtak G, Psaltis AJ, Wormald P-J, Vreugde S. 2023. Pharokka: a fast scalable bacteriophage annotation tool. Bioinformatics 39:btac776. doi:10.1093/bioinformatics/btac776 PMC9805569 36453861

[64] Danecek P, Bonfield JK, Liddle J, Marshall J, Ohan V, Pollard MO, Whitwham A, Keane T, McCarthy SA, Davies RM, Li H. 2021. Twelve years of SAMtools and BCFtools. Gigascience 10:giab008. doi:10.1093/gigascience/giab008 PMC7931819 33590861

[65] Kolmogorov M, Yuan J, Lin Y, Pevzner PA. 2019. Assembly of long, error-prone reads using repeat graphs. Nat Biotechnol 37:540–546. doi:10.1038/s41587-019-0072-8 30936562

[66] Walker BJ, Abeel T, Shea T, Priest M, Abouelliel A, Sakthikumar S, Cuomo CA, Zeng Q, Wortman J, Young SK, Earl AM. 2014. Pilon: an integrated tool for comprehensive microbial variant detection and genome assembly improvement. PLoS One 9:e112963. doi:10.1371/journal.pone.0112963 PMC4237348 25409509

[67] Hunt M, Silva ND, Otto TD, Parkhill J, Keane JA, Harris SR. 2015. Circlator: automated circularization of genome assemblies using long sequencing reads. Genome Biol 16:294. doi:10.1186/s13059-015-0849-0 PMC4699355 26714481

[68] Schwengers O, Jelonek L, Dieckmann MA, Beyvers S, Blom J, Goesmann A. 2021. Bakta: rapid and standardized annotation of bacterial genomes via alignment-free sequence identification. Microb Genomics 7:000685. doi:10.1099/mgen.0.000685 PMC8743544 34739369

[69] Meier-Kolthoff JP, Göker M. 2017. VICTOR: genome-based phylogeny and classification of prokaryotic viruses. Bioinformatics 33:3396–3404. doi:10.1093/bioinformatics/btx440 PMC5860169 29036289

[70] Moraru C, Varsani A, Kropinski AM. 2020. VIRIDIC-a novel tool to calculate the intergenomic similarities of prokaryote-infecting viruses. Viruses 12:1268. doi:10.3390/v12111268 PMC7694805 33172115

[71] Dyer NP, Päuker B, Baxter L, Gupta A, Bunk B, Overmann J, Diricks M, Dreyer V, Niemann S, Holt KE, Rahman M, Brown PE, Stark R, Zhou Z, Ott S, Nübel U. 2025. EnteroBase in 2025: exploring the genomic epidemiology of bacterial pathogens. Nucleic Acids Res 53:D757–D762. doi:10.1093/nar/gkae902 PMC11701629 39441072

[72] Croucher NJ, Page AJ, Connor TR, Delaney AJ, Keane JA, Bentley SD, Parkhill J, Harris SR. 2015. Rapid phylogenetic analysis of large samples of recombinant bacterial whole genome sequences using gubbins. Nucleic Acids Res 43:e15. doi:10.1093/nar/gku1196 PMC4330336 25414349

[73] Page AJ, Taylor B, Delaney AJ, Soares J, Seemann T, Keane JA, Harris SR. 2016. *SNP-sites*: rapid efficient extraction of SNPs from multi-FASTA alignments. Microb Genom 2:e000056. doi:10.1099/mgen.0.000056 PMC5320690 28348851

[74] Nguyen L-T, Schmidt HA, von Haeseler A, Minh BQ. 2015. IQ-TREE: a fast and effective stochastic algorithm for estimating maximum-likelihood phylogenies. Mol Biol Evol 32:268–274. doi:10.1093/molbev/msu300 PMC4271533 25371430

[75] Hoang DT, Chernomor O, von Haeseler A, Minh BQ, Vinh LS. 2018. UFBoot2: improving the ultrafast bootstrap approximation. Mol Biol Evol 35:518–522. doi:10.1093/molbev/msx281 PMC5850222 29077904

[76] Letunic I, Bork P. 2024. Interactive tree of life (iTOL) v6: recent updates to the phylogenetic tree display and annotation tool. Nucleic Acids Res 52:W78–W82. doi:10.1093/nar/gkae268 PMC11223838 38613393

[77] Rambaut A, Lam TT, Max Carvalho L, Pybus OG. 2016. Exploring the temporal structure of heterochronous sequences using TempEst (formerly Path-O-Gen). Virus Evol 2:vew007. doi:10.1093/ve/vew007 PMC4989882 27774300

[78] Wick RR, Judd LM, Gorrie CL, Holt KE. 2017. Unicycler: resolving bacterial genome assemblies from short and long sequencing reads. PLoS Comput Biol 13:e1005595. doi:10.1371/journal.pcbi.1005595 PMC5481147 28594827

[79] Gurevich A, Saveliev V, Vyahhi N, Tesler G. 2013. QUAST: quality assessment tool for genome assemblies. Bioinformatics 29:1072–1075. doi:10.1093/bioinformatics/btt086 PMC3624806 23422339

[80] Chklovski A, Parks DH, Woodcroft BJ, Tyson GW. 2023. CheckM2: a rapid, scalable and accurate tool for assessing microbial genome quality using machine learning. Nat Methods 20:1203–1212. doi:10.1038/s41592-023-01940-w 37500759

[81] Darling AE, Mau B, Perna NT. 2010. progressiveMauve: multiple genome alignment with gene gain, loss and rearrangement. PLoS One 5:e11147. doi:10.1371/journal.pone.0011147 PMC2892488 20593022

[82] Akhter S, Aziz RK, Edwards RA. 2012. PhiSpy: a novel algorithm for finding prophages in bacterial genomes that combines similarity- and composition-based strategies. Nucleic Acids Res 40:e126. doi:10.1093/nar/gks406 PMC3439882 22584627

