# Supplementary material for: Prophage ΦESI promotes competitive dominance in the emergent Salmonella serovar Infantis lineage
Source: Microbiol Spectr. 2026 Sep 25;14(11):e01159-26. doi: 10.1128/spectrum.01159-26 (PMC13729155; doi:10.1128/spectrum.01159-26)
Supplement: Supplementary file 1 [file reviewer-comments.pdf]

ASM | Microbiology Spectrum

Prophage ΦESI promotes competitive dominance in the emergent *Salmonella* serovar Infantis lineage

Cristobal Martinez-Padilla, Daniel Tichy, Rocio Barron-Montenegro, Ignacia Herrera-Vergini, Juan Ugalde, Diana Alvarez, Andrea Moreno Switt, and Alejandro Piña-Iturbe

Corresponding Author(s): Alejandro Piña-Iturbe, Pontificia Universidad Catolica de Chile

Review Timeline:

Submission Date:	April 3, 2026
Editorial Decision:	May 4, 2026
Revision Received:	August 3, 2026
Accepted:	August 28, 2026

Editor: William Calero-Cáceres

Reviewer(s): Disclosure of reviewer identity is with reference to reviewer comments included in decision letter(s). The following individuals involved in review of your submission have agreed to reveal their identity: Shan Goh (Reviewer #1)

Transaction Report:

(Note: With the exception of the correction of typographical or spelling errors that could be a source of ambiguity, letters and reports are not edited. The original formatting of letters and referee reports may not be reflected in this compilation.)

DOI: https://doi.org/10.1128/spectrum.01159-26

Re: Spectrum01159-26 (Prophage ΦESI promotes competitive dominance in the emergent *Salmonella* serovar Infantis lineage)

Dear Dr. Alejandro Piña-Iturbe:

Thank you for the privilege of reviewing your work. Below you will find my comments, instructions from the Spectrum editorial office, and the reviewer comments.

Please return the manuscript within 60 days; if you cannot complete the modification within this time period, please contact me. If you do not wish to modify the manuscript and prefer to submit it to another journal, notify me immediately so that the manuscript may be formally withdrawn from consideration by Spectrum.

Revision Guidelines
To submit your modified manuscript, log into the submission site at https://spectrum.msubmit.net/cgi-bin/main.plex. Go to Author Tasks and click the appropriate manuscript title to begin. The information you entered when you first submitted the paper will be displayed; update this as necessary. Note the following requirements:

• Upload point-by-point responses to the issues raised by the reviewers in a file named "Response to Reviewers," NOT in your cover letter.
• Upload a compare copy of the manuscript (without figures) as a "Marked-Up Manuscript" file.
• Upload a clean .DOC/.DOCX version of the revised manuscript and remove the previous version.
• Each figure must be uploaded as a separate, editable, high-resolution file (TIFF or EPS preferred), and any multipanel figures must be assembled into one file.
• Any supplemental material intended for posting by ASM should be uploaded with their legends separate from the main manuscript. You can combine all supplemental material into one file (preferred) or split it into a maximum of 10 files with all associated legends included.

For complete guidelines on revision requirements, see our Submission and Review Process webpage. Submission of a paper that does not conform to guidelines may delay acceptance of your manuscript.

Data availability: ASM policy requires that data be available to the public upon online posting of the article, so please verify all links to sequence records, if present, and make sure that each number retrieves the full record of the data. If a new accession number is not linked or a link is broken, provide Spectrum production staff with the correct URL for the record. If the accession numbers for new data are not publicly accessible before the expected online posting of the article, publication may be delayed; please contact production staff (Spectrum@asmusa.org) immediately with the expected release date.

Publication Fees: For information on publication fees and which article types are subject to charges, visit our website. If your manuscript is accepted for publication and any fees apply, you will be contacted separately about payment during the production process; please follow the instructions in that e-mail. Arrangements for payment must be made before your article is published.

ASM Membership: Corresponding authors may join or renew ASM membership to obtain discounts on publication fees. Need to upgrade your membership level? Please contact Customer Service at Service@asmusa.org.

The ASM Journals program strives for constant improvement in our submission and publication process. Please tell us how we can improve your experience by taking this quick Author Survey.

Thank you for submitting your paper to Spectrum.

Sincerely,
William Calero-Cáceres
Editor
Microbiology Spectrum

Reviewer #1 (Comments for the Author):

1. Line 131. Could ΦESI be induced chemically or by UV? i.e. the usual means of inducing a prophage? Or is it only induced upon co-culture with another Salmonella Infantis? This would be interesting to state.

2. Line 157-163. The 'translocation' observed may point towards imprecise packaging of concatemeric phage genome into

capsid heads using 'headful packaging' mechanism. That fits with the terminally redundant ends. Did the authors observe concatemeric effects when assembling the phage genomes?

3. Fig 4 and main text associated with it. It is known that phage attachment sites on bacterial genomes have different loci, depending on the genome sequence and the occurrence of the attB sequence. Hence it may not be surprising to see this in different Salmonella Infantis genomes, particularly if isolates were related/clonal. Is there any information on this?
4. Fig 4 and main text associated with it. The 'correlation' of prophage integration and plasmid occurrence is interesting but were there any other (predicted) attB sites in the genomes which the prophage could have used but did not, in the presence of the plasmid?

5. Fig 5 and main text associated with it. Lysogens of ΦESI would be expected to be immune to superinfection (therefore 'fitter' than non-lysogen) by the same or related phage. This is a well-known behaviour in phage and lysogens. It follows that co-culture of lysogens and non-lysogens will result in lysis of non-lysogens with spontaneous prophage induction from the lysogen. Interesting to see that only a relatively small population of lysogenic cells were sacrificed for prophage induction, and the increasing phage from 4-6h likely came more from infection and lysis of DR006 than continued induction of PM57. Would the authors agree?

6. Following on from above, the more interesting questions which the authors should comment on are:
a) co-culture of DR006 and PM57 induced phage (few if any phage was detected in monoculture). What in DR006 is causing this? Simply nutrient competition?
b) whether DR006 became lysogens ΦESI, which would explain its growth in co-culture (hence phage resistance) shown in Fig 5B, C, D.

7. Fig 6 and main text associated. Superinfection immunity relies on relatedness of the prophage and superinfecting phage. It is not surprising to find lysogens highly related to ΦESI are resistant to phage infection while lysogens distantly related were sensitive with high EoP. Other than ΦESI-related CDS, did the authors find any other predicted anti-phage systems in the genomes and pESI?

8. Line 325 'Prophages can substantially limit the spread of conjugative plasmids by killing susceptible transconjugant bacteria, thereby shrinking the pool of viable plasmid recipients'.

I don't entirely agree with this statement because D006 was not completely eliminated by ΦESI and there certainly viable cells present (Fig 5C, D, E). Is it not possible that ΦESI formed new lysogens in some population of DR006 cells so that conjugation of pESI can take place? It seems more likely that ΦESI and pESI are working to benefit each other's spread, similar to a situation reported by Fillol-Salom et al. (2019) PLoS Pathog Jul 5;15(7): e1007888. doi: 10.1371/journal.ppat.1007888 Would this not fit better with your hypothesis in lines 320-322?

9. Line 408. '..and incubated at 37 ºC overnight.' Was that incubated standing or shaking?

10. Line 416. '..fourth passage was stung...' I don't understand the term 'stung'. Do you mean the plaque was picked?

11. qPCR methodology. Please provide details on primer efficiency and how standard curves were generated. i.e. was a recombinant plasmid containing the target sequence created and ran with each qPCR assay? If this assay was established previously, please provide citation.

12. Line 623. PM57_qPCR_Fw and Rev targets the baseplate X gene - is this a prophage sequence? If so, wouldn't the qPCR quantify both PM57 and phage virions produced in co-culture, rather than just the lysogen alone? Why was not a single-copy chromosome associated bacterial sequence used as a target?

13. There were no supplementary material uploaded that I could find, hence I could not refer to them and assess data related to them.

Reviewer #2 (Public repository details (Required)):

Phage genome

Reviewer #2 (Comments for the Author):

The authors put forth an interesting and relevant analysis of phage-bacteria interactions and genome evolution in this problematic foodborne pathogen. There are a number of technical gaps discussed below, though they can be remediated by different analyses of their existing data. With some modification this manuscript will be a strong contribution to the fields of phage biology, genome evolution, and emergent pathogen biology.

The authors put forth an interesting and relevant analysis of phage-bacteria interactions and genome evolution in this problematic foodborne pathogen. There are a number of technical gaps discussed below, though they can be remediated by different analyses of their existing data. With some modification this manuscript will be a strong contribution to the fields of phage biology, genome evolution, and emergent pathogen biology.

Questions and comments

Line 153: Is the integration site of phage ESI conserved in DR 006?

Figure 2B: It would be helpful to include in the alignment the region around the attB in DR006. This would answer the question above.

Line 157-159: This “translocation” is likely an artifact of assembly due to a circular phage genome. If so, you can rotate the phage genome to make it syntenic with the prophage for the purposes of the alignment.

Lines 160-163: This phrasing is confusing. Are the attB, attP, attL, and attR identical? Are they the 15bp or the 127bp sequence? Are the attL and attR direct or indirect?

Lines 163-165: This is not consistent, instead it suggests that your culture conditions allow for lytic replication and plaque formation on DR006. If you were to sequence a population of DR006 after phage addition, there would likely be some rate of integration.

Lines 165-166: You should use your pre-existing sequencing data for the PM57 genome to confirm the prophage is mobilizing via some rate of spontaneous induction. Reads can be mapped to the phage genome and filtered to identify those that span the attP. You can normalize this to reads spanning the attL and attR to quantify an induction rate.

Figure 3A: This is redundant with 3B, which is more intuitive.

Lines 171-173: Be explicit here about the identity cutoffs you used to define “conservation.”

Lines 174-178: Missing a word at each percentage. For example, “39.9% of genomes”

Lines 180-184: Defining phage groups based simply on CDS counts is not a biologically relevant way to group them. Instead, I recommend clustering phages based on their gene content. This will make a more informative analysis when performing correlative analyses with pESI megaplasmids.

Figure 3D: it would be easier to interpret this if you used ESI-like groups (based on clustering) rather than a continuous scale for the number of CDSs. Additionally, there is an innermost ring the is grey throughout.

Fig 4A: Rotation of circular bacterial genomes does not account for genome rearrangement, and this analysis is not informative. You later account for genome rearrangement, which is significantly more meaningful.

Lines 212-226: Using the coordinates to describe the groups is overwhelming. Simplify this in the writing and instead focus of the genome rearrangment itself (shown in the Fig 4C) and leave it to the figure (already shown in blue/yellow). This will make it easier for your reader to ascertain which prophages are complete (and more likely activate).

Fig 4C: I love this figure except for the way connectors are shown. I see that regions that are moved are in red and conserved ones in grey. It would be immensely helpful to uniquely color connectors for each region, making this more readable.

Fig 4D: Find a different way to better show these associations. The Sankey diagram is not appropriate here, and also has too much information embedded for your ultimate conclusion about profile-continent correlation.
Line 241: Be explicit that this advantage is in vitro.

Line 245… Figure 5: Reorder figure subsections to match their initial occurrence in the manuscript (for example, you refer to 5C before 5B).

Fig 5G: Make a quantitative chart rather than pictures of plaques. It is easier to read.

Lines 268-270: If you perform the above analysis about spontaneous induction, it would be valuable to put forth a biological hypothesis/interpretation of the data: (1) phage induction from PM57 does not affect its population's growth, (2) non-induced PM57 subpopulation is resistant to new phage infection and lysis, (3) only a subpopulation of DR006 is killed by phage ESI, as there is still a lot of growth seen.

Lines 275-277: How is it that there is no correlation between phage ESI and pESI in your collection, but there was an enormously significant correlation in earlier analyses? This is very concerning.

Lines 278-285: What genes? Again, this would SIGNIFICANTLY benefit from clustering based on gene content as described above. Once you have newly described groups, you can use those here. This will also enable an analyses of WHICH gene(s) that confer superinfection immunity (Line 285). Another easy analysis (assuming you have complete genomes for these 19 strains) would be to look for non-phage factors that inhibit infection.

Discussion: Be clear if this work is simply observational (not a bad thing!) or could be applied to remediating Si emergence.

Point-by-point response

We appreciate the time and effort made by the reviewers in handling our manuscript and appreciate their feedback. All comments were addressed and below is a detailed response to each one. Changes in the manuscript are indicated with the line number and highlighted according to each reviewer's color.

Reviewer 1

1. Line 131. Could ΦESI be induced chemically or by UV? i.e. the usual means of inducing a prophage? Or is it only induced upon co-culture with another Salmonella Infantis? This would be interesting to state.

To test induction by a chemical with real-world relevance, we tested the role of peracetic acid (PAA), an organic acid found by *Salmonella* Infantis during processing of chicken meat. We determined the MIC of PAA in TSB medium (MIC= 39 ppm) and then tested whether the challenge of strain PM57 with subinhibitory concentrations of PAA could increase the plaque count in monoculture. We found no difference between the PFU count in the supernatant of the PAA-treated vs the control (one-way ANOVA). We have also carried out assays to test the effect of a nutrient-depleted medium and of putative DR006-derived secreted molecules as part of the response to Comment 6 (please, see below).

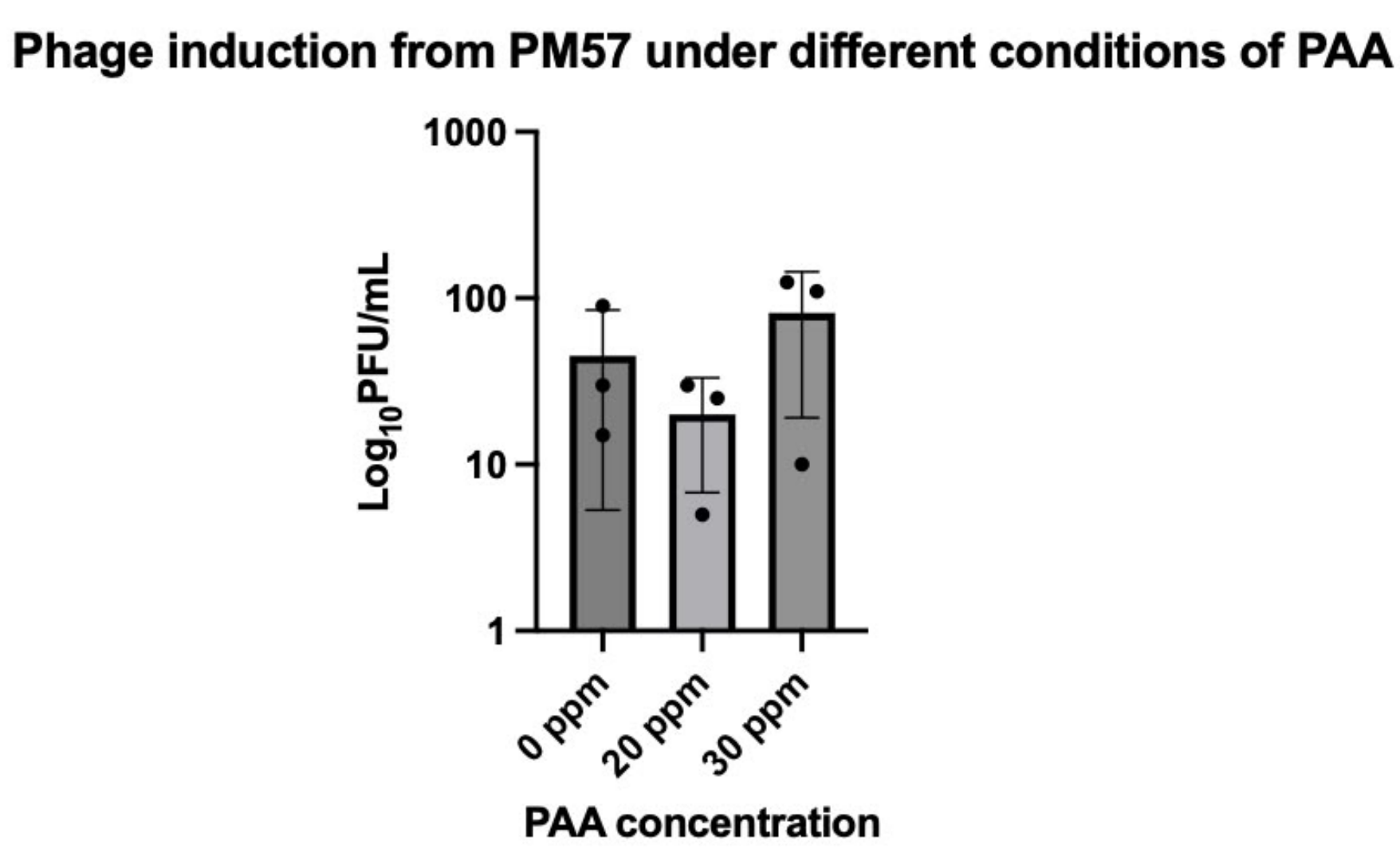

2. Line 157-163. The 'translocation' observed may point towards imprecise packaging of concatemeric phage genome into capsid heads using 'headful packaging' mechanism. That fits with the terminally redundant ends. Did the authors observe concatemeric effects when assembling the phage genomes?

The "translocation" mentioned in the text was intended to describe how gene synteny in prophage vs virion was affected as the result of phage genome integration. The text in the mentioned lines has been rewritten for clarity, and also in response to Reviewer 2. Lines 159-168.

We also appreciate the reviewer's question. Phage genome sequencing did not reveal evidence for concatemeric phage DNA. The SPAdes assembly produced a phage contig of 46.5 kb containing identical 127-bp terminal repeats at both ends, and no tandem genome copies, oversized contigs, or internal genome–genome junctions indicative of concatemeric assembly were detected.

3. Fig 4 and main text associated with it. It is known that phage attachment sites on bacterial genomes have different loci, depending on the genome sequence and the occurrence of the attB sequence. Hence it may not be surprising to see this in different Salmonella Infantis genomes, particularly if isolates were related/clonal. Is there any information on this?

We thank the reviewer's comment. We found that, although there are 7 Arg-tRNA in the *Salmonella* Infantis chromosome, the one used as the integration site for phiESI, and in particular the 15bp-long *attB* site at the 3'-end of that tRNA, is unique in each chromosome. However, we also found that for some chromosomes, the *attB* site is either at the 1.5 Mbp loci or at the 3.05 Mbp loci. This is explained by the large-scale inversion of the intervening region between these two loci, observed in 39% of the closed chromosomes harboring phiESI. Therefore, we have modified the text in lines 214-218, 1098-1099, and Fig. 4A to also show the location of the *attB* sequence and better explain the observed features.

4. Fig 4 and main text associated with it. The 'correlation' of prophage integration and plasmid occurrence is interesting but were there any other (predicted) attB sites in the genomes which the prophage could have used but did not, in the presence of the plasmid?

The 15bp-long attB site at the 3'-end of that tRNA, are unique in each chromosome. Therefore, when present, phiESI is inserted at the same integration site. As part of the response to the previous comment, we have modified the text to include a mention of the unique *attB* site. Lines 165-168, 214-218.

5. Fig 5 and main text associated with it. Lysogens of ΦESI would be expected to be immune to superinfection (therefore 'fitter' than non-lysogen) by the same or related phage. This is a well-known behaviour in phage and lysogens. It follows that co-culture of lysogens and non-lysogens will result in lysis of non-lysogens with spontaneous prophage induction from the lysogen. Interesting to see that only a relatively small population of lysogenic cells were sacrificed for prophage induction, and the increasing phage from 4-6h likely came more from infection and lysis of DR006 than continued induction of PM57. Would the authors agree?

We agree with this interpretation of the data. Moreover, we have revised the corresponding text (lines 311–315) to state this point more clearly.

6. Following on from above, the more interesting questions which the authors should comment on are:

a) co-culture of DR006 and PM57 induced phage (few if any phage was detected in monoculture). What in DR006 is causing this? Simply nutrient competition?

We tested the phage-induction capacity of the cell-free supernatant (TSB medium) in which DR006 was grown. We inoculated the supernatant with strain PM57, replicating the conditions of the coculture experiments, and then performed PFU counts. We also included fresh TSB and TSB in which PM57 had grown, as controls. We detected PFU at 3h after incubation at low titers in each of the three tested conditions. No statistically significant difference was observed. We have included these results as Supplementary Figure S6, and in lines 315-316.

b) whether DR006 became lysogens ΦESI, which would explain its growth in co-culture (hence phage resistance) shown in Fig 5B, C, D.

We generated two DR006-derivatives with resistance to the ΦESI-mediated lysis. Both isolates were sequenced and the reads analyzed and uploaded to the SRA. SNP analysis and whole genome global alignments revealed the absence of an integrated ΦESI, but a single nucleotide substitution (in both mutant strains) that was absent in the wildtype control subjected to the same treatments except for the ΦESI challenge. The identified SNP mapped to the *rfbB* gene, causing a frameshift and a premature stop. This gene is part of the operon involved in synthesis of the O-antigen, therfore suggesting that under our *in vitro* conditions, the DR006 strain overcame ΦESI-mediated lysis by receptor mutation. We have modified the manuscript to include the methodological details, and the corresponding discussion, in lines 318-326, 683-718, and 775-777. The *rfbB* alignment was included as Supplementary Figure S7.

7. Fig 6 and main text associated. Superinfection immunity relies on relatedness of the prophage and superinfecting phage. It is not surprising to find lysogens highly related to ΦESI are resistant to phage infection while lysogens distantly related were sensitive with high EoP. Other than ΦESI-related CDS, did the authors find any other predicted anti-phage systems in the genomes and pESI?

We carried out an *in silico* search of anti-phage systems using PADLOC v2. We found a set of conserved defense systems across the 19 lysis-resistant and susceptible strains, and a subset of predicted systems present in some of the ΦESI-carrying isolates, and other systems in the isolates carrying few ΦESI genes (23 CDSs). Therefore, while there was not a pattern of conserved defense systems explaining the lysis resistance, we now acknowledge in the Discussion section that we cannot discard a potential role of particular defense systems on the resistance observed in the isolates harboring 23 CDSs only. Lines 336-342 and Suppl Table S9.

8. Line 325 'Prophages can substantially limit the spread of conjugative plasmids by killing susceptible transconjugant bacteria, thereby shrinking the pool of viable plasmid recipients'.

I don't entirely agree with this statement because D006 was not completely eliminated by ΦESI and there certainly viable cells present (Fig 5C, D, E). Is it not possible that ΦESI formed new lysogens in some population of DR006 cells so that conjugation of pESI can take place? It seems more likely that ΦESI and pESI are working to benefit each other's spread, similar to a situation reported by Fillol-Salom et al. (2019) PLoS Pathog Jul 5;15(7): e1007888. doi: 10.1371/journal.ppat.1007888 Would this not fit better with your hypothesis in lines 320-322?

We thank the reviewer's comment. After considering the points mentioned in the comment. We decided to stick to our original argument mainly because i) the analysis of lysis-resistant mutants (response to comment 6) showed no prophage integration, and ii) the pESI-megaplasmid distribution among *Salmonella* Infantis shows that no other Infantis lineage, besides the emergent lineage, have acquired this conjugative element, even when several other *Salmonella* serovars have received pESI and are ΦESI-negative (unpublished data). We have modified the text to emphasize that plasmid acquisition has occurred by other non-Infantis serovars but not but other Infantis lineages. Lines 359-361.

9. Line 408. '..and incubated at 37 ºC overnight.' Was that incubated standing or shaking?

As requested, "without shaking" was added in Line 447.

10. Line 416. '..fourth passage was stung...' I don't understand the term 'stung'. Do you mean the plaque was picked?

As requested, the word was corrected. Line 455.

11. qPCR methodology. Please provide details on primer efficiency and how standard curves were generated. i.e. was a recombinant plasmid containing the target sequence created and ran with each qPCR assay? If this assay was established previously, please provide citation.

Location of primer hybridization and qPCR efficiency and standard curves are indicated in Figures S10 and S11 in the "SupplementaryFigures.docx" file.

12. Line 623. PM57_qPCR_Fw and Rev targets the baseplate X gene - is this a prophage sequence? If so, wouldn't the qPCR quantify both PM57 and phage virions produced in co-culture, rather than just the lysogen alone? Why was not a single-copy chromosome associated bacterial sequence used as a target?

We thank the reviewer's observation. The only genomic differences found between DR006 and PM57 (BLASTn comparisons of assembled genomes) were prophage ΦESI, the pESI megaplasmid, and the region used for qPCR. WGS of plaque isolated from a second independent DR006-PM57 coculture resulted in re-isolation of ΦESI, indicating that the main amplified phage in the co-coculture is ΦESI. We have added this information in lines 401-413, and 665-667.

13. There were no supplementary material uploaded that I could find, hence I could not refer to them and assess data related to them.

The supplementary files SupplementaryFigures.docx and SupplementaryTables.xlsx were available through the link in line 759, in the Data Availability section.

Reviewer 2:

The authors put forth an interesting and relevant analysis of phage-bacteria interactions and genome evolution in this problematic foodborne pathogen. There are a number of technical gaps discussed below, though they can be remediated by different analyses of their existing data. With some modification this manuscript will be a strong contribution to the fields of phage biology, genome evolution, and emergent pathogen biology.

Questions and comments

1. Line 153: Is the integration site of phage ESI conserved in DR006?

The integration site (the last 15bp of a tRNA-Arg gene) is conserved in DR006 and is empty. We have modified the text to make this explicit. Lines 165-168.

2. Figure 2B: It would be helpful to include in the alignment the region around the attB in DR006. This would answer the question above.

As required, the panel 2B was modified to include the comparison with DR006 which has an empty integration site. Fig 2 and legend in Lines 1071, 1074-1075.

3. Line 157-159: This "translocation" is likely an artifact of assembly due to a circular phage genome. If so, you can rotate the phage genome to make it syntenic with the prophage for the purposes of the alignment.

Changes were made to provide a clearer description of the prophage organization and comparison with the virion DNA, the lines regarding the "translocation" were removed. Lines 159-165.

4. Lines 160-163: This phrasing is confusing. Are the attB, attP, attL, and attR identical? Are they the 15bp or the 127bp sequence? Are the attL and attR direct or indirect?

As required, we have modified the text to answer these questions and provide a clearer presentation of the characteristics of these sequences. Lines 159-168.

5. Lines 163-165: This is not consistent, instead it suggests that your culture conditions allow for lytic replication and plaque formation on DR006. If you were to sequence a population of DR006 after phage addition, there would likely be some rate of integration.

We generated two DR006-derivatives with resistance to the ΦESI-mediated lysis. Both isolates were sequenced and the reads analyzed and uploaded to the SRA. SNP analysis and whole genome global alignments revealed the absence of an integrated ΦESI, but a single nucleotide substitution (in both mutant strains) that was absent in the wildtype control subjected to the same treatments except for the ΦESI challenge. The identified SNP mapped to the *rfbB* gene, causing a frameshift and a premature stop.

This gene is part of the operon involved in synthesis of the O-antigen, therfore suggesting that under our *in vitro* conditions, the DR006 strain overcame ΦESI-mediated lysis by receptor mutation. We have modified the manuscript to include the methodological details, and the corresponding discussion, in lines 318-326, 683-718, and 775-777. The *rfbB* alignment was included as Supplementary Figure S7.

6. Lines 165-166: You should use your pre-existing sequencing data for the PM57 genome to confirm the prophage is mobilizing via some rate of spontaneous induction. Reads can be mapped to the phage genome and filtered to identify those that span the attP. You can normalize this to reads spanning the attL and attR to quantify an induction rate.

We mapped Illumina reads from whole genome sequencing of PM57. However, unexpectedly, no read spanned the empty attB site. Conversely, reads including the attB 15bp-sequence abruptly ended in one end or the other (see figure below).

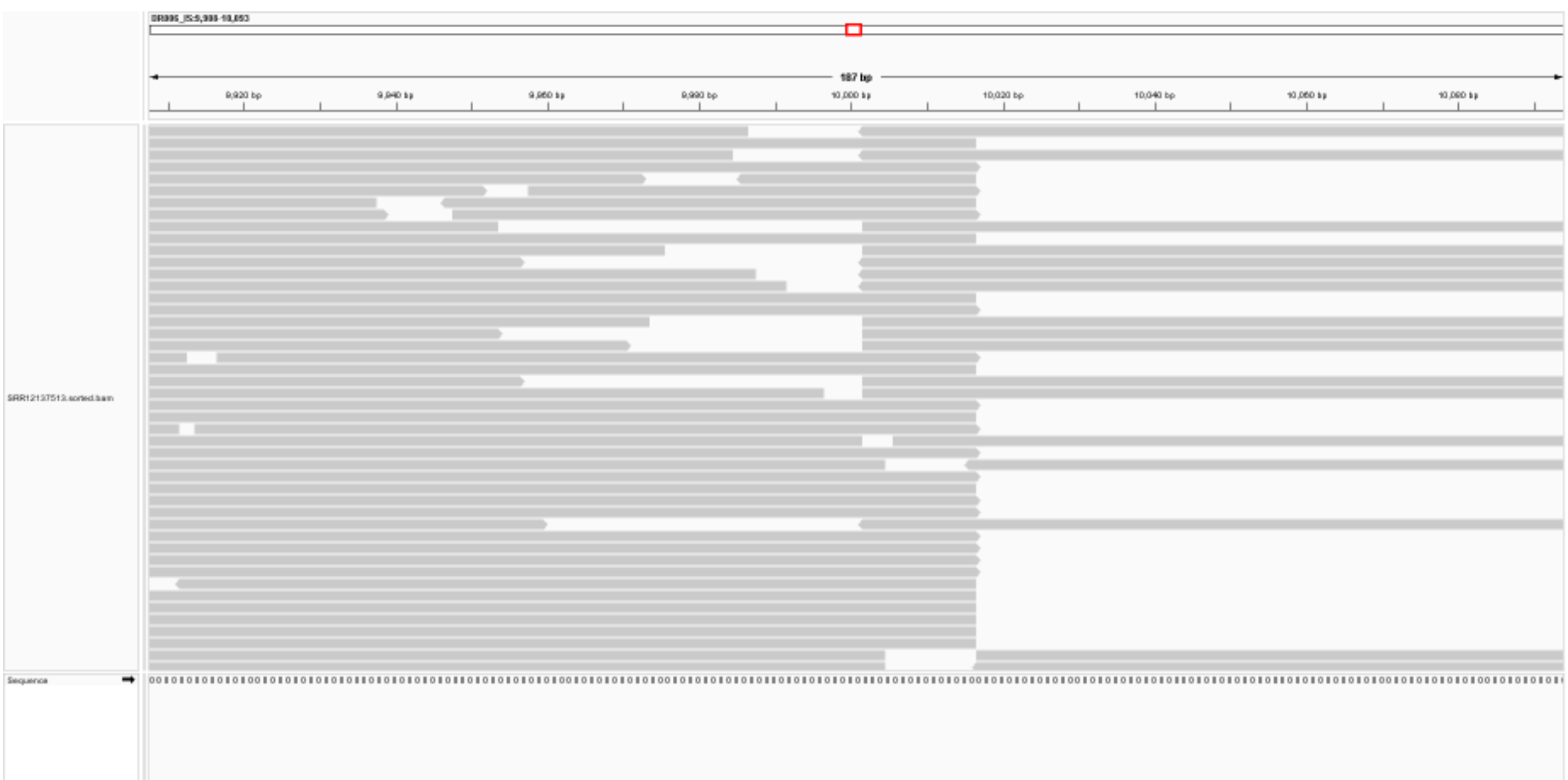

7. Figure 3A: This is redundant with 3B, which is more intuitive.

We have modified Figure 3A to include a heatmap showing the presence/absence, similarity, and gene synteny information of the ΦESI CDSs across the global non-redundant *Salmonella* Infantis genomes analyzed, as part of the response to comment 10 and 11 of the reviewer (please, see below).

8. Lines 171-173: Be explicit here about the identity cutoffs you used to define "conservation."

As requested, the cutoffs of the search (80% identity and coverage) were added to the text. Line 178.

9. Lines 174-178: Missing a word at each percentage. For example, "39.9% of genomes"

We have modified these lines in response to the comments 10 and 11, and as requested, the word "genomes" was added to clearly state what the percentages represent. Lines 181-189.

10. Lines 180-184: Defining phage groups based simply on CDS counts is not a biologically relevant way to group them. Instead, I recommend clustering phages based on their gene content. This will make a more informative analysis when performing correlative analyses with pESI megaplasmids.

As requested, we analyzed the ΦESI CDSs distribution considering their presence/absence, similarity (the product of identity and coverage) and their synteny relative to the virion genome. We have modified our grouping based on the results of this analysis and included a new Fig. 3A, and a modified text and Suppl. Table S4. Lines 174-189, 575-578, 1078-1086.

11. Figure 3D: it would be easier to interpret this if you used ESI-like groups (based on clustering) rather than a continuous scale for the number of CDSs. Additionally, there is an innermost ring the is grey throughout.

We have modified Fig 3D according to the reviewer's suggestions and the groups based on clustering. The "innermost ring" corresponds to the isolates Enterobase assembly barcodes. These modifications are accompanied by text modifications in lines 1093-1094.

12. Fig 4A: Rotation of circular bacterial genomes does not account for genome rearrangement, and this analysis is not informative. You later account for genome rearrangement, which is significantly more meaningful.

With "rotation" we tried to refer to genome reorientation if necessary to normalize the start position (at *dnaN*) and to match the reference orientation. This was carried out to enable position-based comparisons of the loci harboring ΦESI sequences. We have modified the text to convey the right ideas. Lines 205-206. Additionally, in response to comments of reviewer 1, we added the location of the phage insertion sites (*attB*) in Fig. 4A.

13. Lines 212-226: Using the coordinates to describe the groups is overwhelming. Simplify this in the writing and instead focus of the genome rearrangment itself (shown in the Fig 4C) and leave it to the figure (already shown in blue/yellow). This will make it easier for your reader to ascertain which prophages are complete (and more likely activate).

We thank the reviewer's comment. We have simplified the text in the manuscript to provide an overview of the general location patters of the ΦESI-related sequences. Lines 222-231.

14. Fig 4C: I love this figure except for the way connectors are shown. I see that regions that are moved are in red and conserved ones in grey. It would be immensely helpful to uniquely color connectors for each region, making this more readable.

As requested, we tried to apply a color code for each region. However, as there are several rearrangements shown, the result seemed confusing, as some share regions ended with a different coloring because of rearrangements (see below). We opted to keep the original coloring.

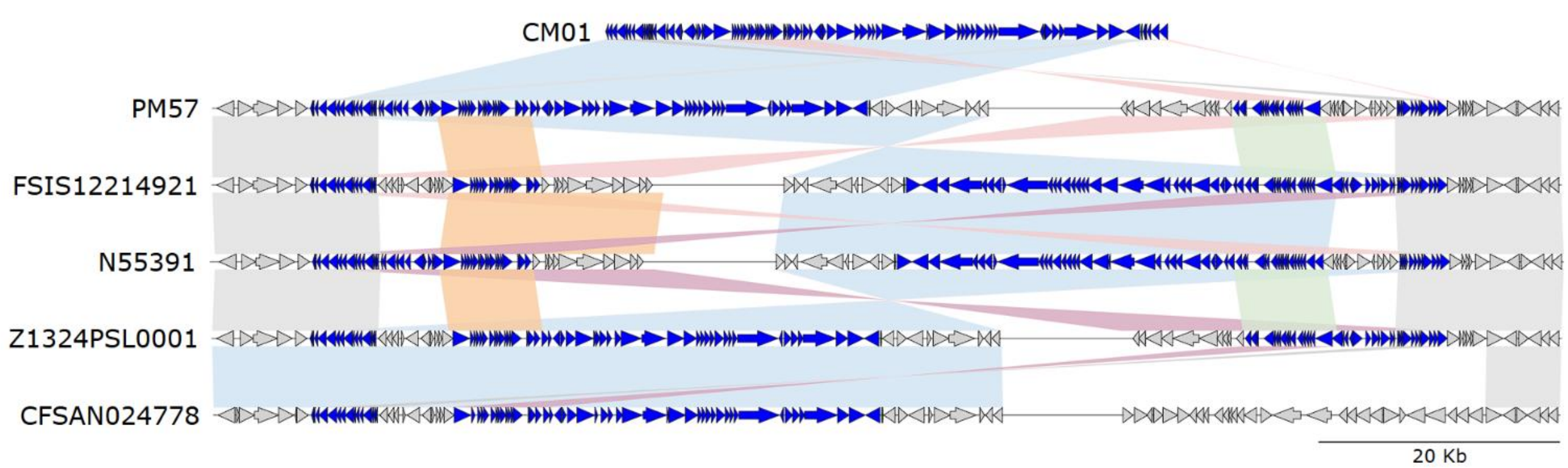

15. Fig 4D: Find a different way to better show these associations. The Sankey diagram is not appropriate here, and also has too much information embedded for your ultimate conclusion about profile-continent correlation.

We evaluated different ways to show the information of panel 4D (an example below). However, after consideration, we opted to keep the original panel since the original purpose was to show the analysis of the distribution of complete chromosome profiles. We have modified the text in lines 237-238 to better cite panel 4D.

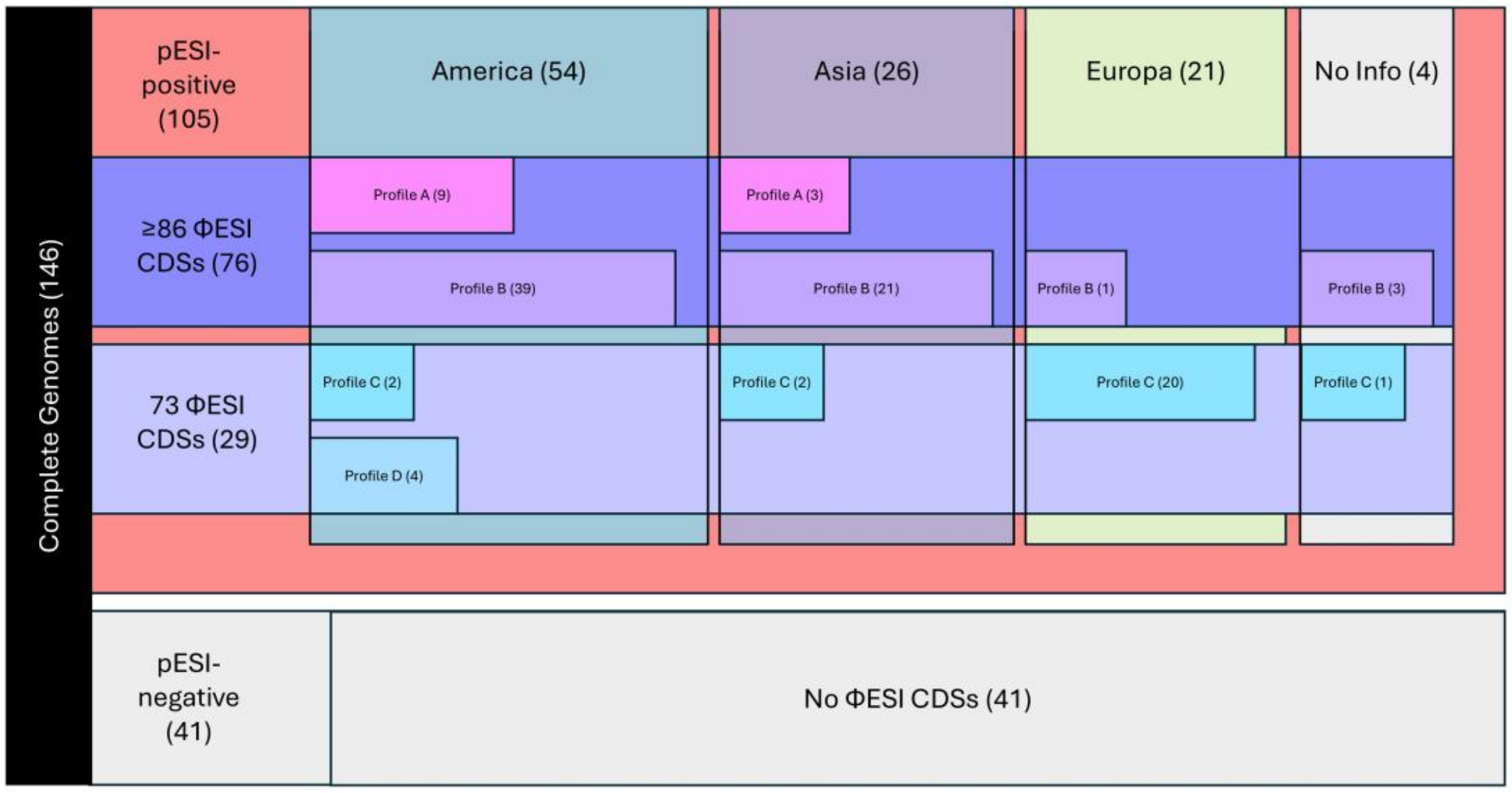

16. Line 241: Be explicit that this advantage is in vitro.

As requested, we have added "*in vitro*". Line 248.

17. Line 245... Figure 5: Reorder figure subsections to match their initial occurrence in the manuscript (for example, you refer to 5C before 5B).

The text in Lines 251-257 was modified to present the same results but correcting for the order of the figure panels presentation.

18. Fig 5G: Make a quantitative chart rather than pictures of plaques. It is easier to read.

As requested, we have added a quantitative graph in panel 5G, and modified figure legend in lines 1127-1128.

19. Lines 268-270: If you perform the above analysis about spontaneous induction, it would be valuable to put forth a biological hypothesis/interpretation of the data: (1) phage induction from PM57 does not affect its population's growth, (2) non-induced PM57 subpopulation is resistant to new phage infection and lysis, (3) only a subpopulation of DR006 is killed by phage ESI, as there is still a lot of growth seen.

We thank the reviewer for this comment. We have included our data interpretation (which is in line with the reviewer's suggestion) on Lines 312-319 of the discussion section.

20. Lines 275-277: How is it that there is no correlation between phage ESI and pESI in your collection, but there was an enormously significant correlation in earlier analyses? This is very concerning.

We appreciate the reviewer's comment. The text "We could not find pESI+/ΦESI- nor pESI+/ΦESI-like+ combinations among our sequenced *Salmonella* collection of more than 2,800 isolates" refers to our laboratory culture collection, which consists of *Salmonella* isolated from Chile. We have corrected the number, as ≈2800 is the number of isolates including all serovars, but serovar Infantis represented 229 isolates only. In this collection, we could not find pESI+/ΦESI- nor pESI+/ΦESI-like+ isolates, which agrees with the global analysis. This is because our collection represents South American (Chilean in particular) isolates that, as observed in the new version of Fig. 3D, almost uniquely belong to the ΦESI group. Representatives of ΦESI-like in *Salmonella* Infantis from the Americas are relatively scarce (Fig. 3D) and were not present in our collection.

We have corrected the number of isolate in our collection and changed the wording in lines 281-284, 724 to state that they do not represent the "complete" diversity of pESI/ΦESI combinations. We have also changed "ΦESI-" with "ΦESI-negative" to avoid confusion.

21. Lines 278-285: What genes? Again, this would SIGNIFICANTLY benefit from clustering based on gene content as described above. Once you have newly described groups, you can use those here. This will also enable an analyses of WHICH gene(s) that confer superinfection immunity (Line 285). Another easy analysis (assuming you have complete genomes for these 19 strains) would be to look for non-phage factors that inhibit infection.

We thank the reviewer's comment. We have modified the text to indicate that the "few genes" are 23. As these are too many genes to mention in the text, we have appended that information in TableS8 which presents the data related to the 19 isolates used in the experiment. We have also carried out *in silico* search of anti-phage systems with PADLOC. Finally, the discussion regarding the role of the potential genes involved in superinfection exclusion and the anti-phage systems is in Lines 291, 330-343of the Discussion section.

22. Discussion: Be clear if this work is simply observational (not a bad thing!) or could be applied to remediating Si emergence.

We thank the reviewer's comment. The modifications we have made to the Discussion section have underscored the observational nature of our work.

Re: Spectrum01159-26R1 (Prophage ΦESI promotes competitive dominance in the emergent *Salmonella* serovar Infantis lineage)

Dear Dr. Alejandro Piña-Iturbe:

Your manuscript has been accepted, and I am forwarding it to the ASM production staff for publication. Your paper will first be checked to make sure all elements meet the technical requirements. ASM staff will contact you if anything needs to be revised before copyediting and production can begin. Otherwise, you will be notified when your proofs are ready to be viewed.

Data Availability: ASM policy requires that data be available to the public upon online posting of the article, so please verify all links to sequence records, if present, and make sure that each number retrieves the full record of the data. If a new accession number is not linked or a link is broken, provide production staff with the correct URL for the record. If the accession numbers for new data are not publicly accessible before the expected online posting of the article, publication may be delayed; please contact ASM production staff immediately with the expected release date.

Publication Fees: For information on publication fees and which article types have charges, please visit our website. We have partnered with Copyright Clearance Center (CCC) to collect author charges. If fees apply to your paper, you will receive a message from no-reply@copyright.com with further instructions. For questions related to paying charges through RightsLink, please contact CCC at ASM_Support@copyright.com or toll free at +1-877-622-5543. CCC makes every attempt to respond to all emails within 24 hours.

ASM Membership: Corresponding authors may join or renew ASM membership to obtain discounts on publication fees. Need to upgrade your membership level? Please contact Customer Service at Service@asmusa.org.

Embargo Policy: A press release may be issued as soon as the manuscript is posted on the Spectrum Latest Articles webpage. The corresponding author will receive an email with the subject line "ASM Journals Author Services Notification" when the article is available online.

Cover Image Submissions: If you would like to submit a potential Cover Image, please email a file and a short legend to spectrum@asmusa.org. Please note that we can only consider images that (i) the authors created or own and (ii) have not been previously published. By submitting, you agree that the image can be used under the same terms as the published article. Image File requirements: TIF/EPS, 7.5 inches wide by 8.25 inches tall (at least 2,250 pixels wide by 2,475 pixels tall), minimum 300 dpi resolution (600 dpi preferred), RGB, and no figure elements, e.g., arrows or panel labels. The legend should be a short description of the image, 1-2 sentences recommended. Please use the ASM Journals Cover Image Sizing Tool (https://journals.asm.org/pb-assets/html/asm-cover-sizing-tool.html) to ensure that your proposed cover image meets our size requirements.

The ASM Journals program strives for constant improvement in our submission and publication process. Please tell us how we can improve your experience by taking this quick Author Survey.

Thank you for submitting your paper to Spectrum.

Sincerely,
William Calero-Cáceres
Editor
Microbiology Spectrum

Reviewer #1 (Comments for the Author):

Thanks for thoroughly addressing my concerns!

Reviewer #2 (Comments for the Author):

I thank the authors for their thoughtful edits and responses.
